# Sonodynamic Therapy of NRP2 Monoclonal Antibody‐Guided MOFs@COF Targeted Disruption of Mitochondrial and Endoplasmic Reticulum Homeostasis to Induce Autophagy‐Dependent Ferroptosis

**DOI:** 10.1002/advs.202303872

**Published:** 2023-09-03

**Authors:** Zhiyu Zhao, Yanjie Wu, Xiaochen Liang, Jiajing Liu, Yi Luo, Yijia Zhang, Tingting Li, Cong Liu, Xian Luo, Jialin Chen, Yunjie Wang, Shengyu Wang, Ting Wu, Shaoliang Zhang, Dong Yang, Wengang Li, Jianghua Yan, Zhihai Ke, Fanghong Luo

**Affiliations:** ^1^ Cancer Research Center School of Medicine Xiamen University Xiamen 361000 P.R. China; ^2^ School of Science and Engineering Shenzhen Key Laboratory of Innovative Drug Synthesis The Chinese University of Hong Kong Shenzhen 518172 P.R. China; ^3^ Environmental Toxicology University of California Riverside California 92507 USA; ^4^ School of Basic Medicine School of Clinical Medicine Fujian Medical University Fuzhou 350122 P.R. China; ^5^ Shanghai Guangsheng Biopharmaceutical Co., Ltd Shanghai 200120 P.R. China

**Keywords:** covalent organic framework (COF), endoplasmic Reticulum (ER) stress, ferroptosis, gemcitabine, metal organic framework (MOFs), sonodynamic therapy

## Abstract

The lethality and chemotherapy resistance of pancreatic cancer necessitates the urgent development of innovative strategies to improve patient outcomes. To address this issue, we designed a novel drug delivery system named GDMCN2,which uses iron‐based metal organic framework (Fe‐MOF) nanocages encased in a covalent organic framework (COF) and modified with the pancreatic cancer‐specific antibody, NRP2. After being targeted into tumor cells, GDMCN2 gradually release the sonosensitizer sinoporphyrin sodium (DVDMS) and chemotherapeutic gemcitabine (GEM) and simultaneously generated reactive oxygen species (ROS) under ultrasound (US) irradiation. This system can overcome gemcitabine resistance in pancreatic cancer and reduce its toxicity to non‐targeted cells and tissues. In a mechanistic cascade, the release of ROS activates the mitochondrial transition pore (MPTP), leading to the release of Ca^2+^ and induction of endoplasmic reticulum (ER) stress. Therefore, microtubule‐associated protein 1A/1B‐light chain 3 (LC3) is activated, promoting lysosomal autophagy. This process also induces autophagy‐dependent ferroptosis, aided by the upregulation of Nuclear Receptor Coactivator 4 (NCOA4). This mechanism increases the sensitivity of pancreatic cancer cells to chemotherapeutic drugs and increases mitochondrial and DNA damage. The findings demonstrate the potential of GDMCN2 nanocages as a new avenue for the development of cancer therapeutics.

## Graphical abstract

Researchers have developed a new drug delivery system for pancreatic cancer treatment. The GDMCN2 system consists of MOFs enclosed in a COF and modified with the pancreatic cancer‐specific antibody NRP2. GDMCN2 gradually releases the sonosensitizers DVDMS and gemcitabine while generating ROS under US irradiation. This approach overcomes GEM resistance, reduces toxicity, and enhances the sensitivity to chemotherapy. These findings highlight the potential of GDMCN2 as a therapeutic target in pancreatic cancer.

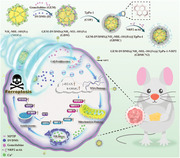

## 1 Introduction

Pancreatic cancer is a highly aggressive malignancy with a poor prognosis; most patients are diagnosed at advanced stages, resulting in limited options for surgical intervention.^[^ ^1^ ^]^ Chemotherapy is the primary treatment for advanced pancreatic cancer, with GEM as the first‐line drug; however, its efficacy is often limited due to drug resistance.^[^ ^1^, ^2^ ^]^ Sonodynamic therapy (SDT), which employs high‐energy sound waves to generate mechanical vibrations, has been widely used to treat solid tumors, such as liver and kidney cancers.^[^ ^3^ ^]^ However, its application in pancreatic cancer is limited primarily because of drug resistance. Recent research has indicated that SDT is a promising option for treating pancreatic cancer; however, effective drug carriers are urgently required to enhance its efficacy and overcome drug resistance.^[^ ^4^ ^]^

Over the past few decades, various nanocarriers have been developed to enhance drug solubility, stability, and concentration.^[^ ^5^ ^]^ However, they have drawbacks, such as limited loading capacity, undesirable toxicity, and unacceptable biodegradability.^[^ ^6^ ^]^ Recently, metal organic framework (MOFs) comprising inorganic metal subunits and organic ligands have emerged as promising carrier materials. Unlike traditional inorganic carriers, MOFs exhibit enhanced biodegradability because of the presence of organic ligands.^[^ ^7^ ^]^ Within biological systems, MOFs undergo degradation reactions and gradually transform into harmless metabolites, thereby mitigating concerns regarding their long‐term accumulation and potential toxicity.^[^ ^8^ ^]^ This unique characteristic makes MOFs attractive choices for various biomedical applications.^[^ ^9^ ^]^ Additionally, MOFs have garnered significant attention owing to their exceptional features, including a high specific surface area, tunable pore size, and structural stability.^[^ ^6^, ^10^ ^]^ Fe‐MOFs have attracted considerable interest owing to their photophysical properties, low toxicity, structural stability, and adaptable surface functionalities.^[^ ^11^ ^]^ Fe‐MOFs damage mitochondria and DNA, resulting in cell death.^[^ ^12^ ^]^ Recent research has indicated that Fe‐MOFs can induce tumor cells to undergo ferroptosis, a novel mode of cell death characterized by lipid peroxide accumulation and excess iron, making them an attractive option for GEM‐resistant tumors.^[^ ^13^ ^]^

The relatively poor hydrophilicity of many MOFs affects drug release and delivery in vivo.^[^ ^14^ ^]^ Consequently, covalent organic framework (COF), synthesized via the Schiff base reaction of 2,4,6‐triformylphloroglucinol (Tp) with p‐phenylenediamine (Pa‐1), have been reported to exhibit remarkable hydrolytic stability of MOFs in water.^[^ ^15^ ^]^ COF possesses good biodegradability resulting from reversible dynamic linkages. Thus, these linkages can break in acid, enabling the pH‐triggered release of the drug.^[^ ^16^ ^]^ Thus, we rationalized that the hydrophilic TpPa‐1 COF was prepared as a shell layer to reduce drug leakage from the GEM‐DVDMS@NH_2_‐MIL‐101(Fe) composite. Moreover, the hydrophilic pore environment favors the maintenance of the native conformation of the protein, making it ideal for conjugating antibodies.

NRP2, a cell surface molecule widely present in pancreatic cancer cells, plays a crucial role in tumor cell growth, migration, invasion, and angiogenesis.^[^ ^17^ ^]^ Monoclonal antibodies against NRP2 have been proposed as a potential strategy for pancreatic cancer treatment. A mouse monoclonal antibody (mAb) was developed to specifically bind to pancreatic ductal adenocarcinoma cells.^[^ ^18^ ^]^ By linking the NRP2 mAb to the surface of the GEM‐DVDMS@NH_2_‐MIL‐101(Fe) composite, tumor targeting was improved, enabling the drug system to quickly reach the tumor site. Further research is needed to explore the mechanisms of action, efficacy, and safety of this promising drug delivery system.

**Scheme 1:**
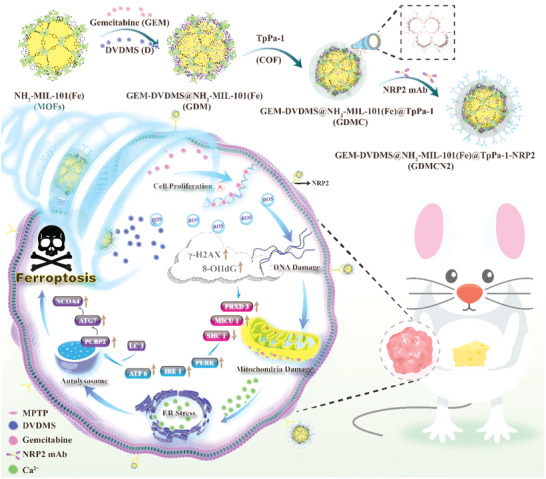
SDT of NRP2 mAb‐guided MOFs@COF targeted disruption of mitochondrial and ER homeostasis to induce autophagy‐dependent ferroptosis.

To address the issue of GEM‐resistant pancreatic cancer, we designed a novel nanocage, GEM‐DVDMS@MOFs@COF‐NRP2 (GDMCN2), which specifically targets human GEM‐resistant cells (PANC‐1/GEM) via pancreatic cancer‐specific targeting. Upon sonodynamic stimulation, GDMCN2 generates a substantial amount of reactive oxygen species (ROS) that induces mitochondrial and DNA damage in cells. This leads to the activation of ER stress and ultimately triggers autophagy‐dependent ferroptosis. This strategy has the potential to completely eradicate pancreatic cancer tumors and provides a potentially efficient therapeutic approach for the clinical management of GEM‐resistant pancreatic cancer (**Scheme** **1**).

## 2 Results and Discussion

### 2.1 Synthesis and Characterization of GDMCN2

**Figure 1:**
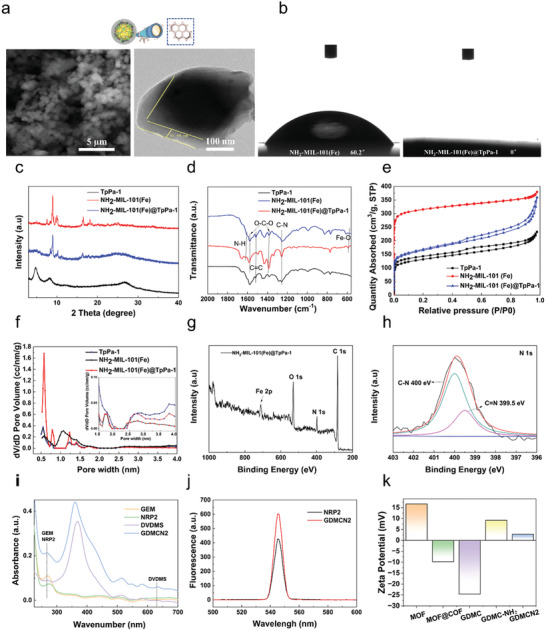
a) SEM TEM images of GEM‐DVDMS@NH_2_‐MIL‐101 (Fe)@TpPa‐1. b) The water contact angle of NH_2_‐MIL‐101(Fe) and NH_2_‐MIL‐101(Fe)@TpPa‐1 c) XRD patterns of TpPa‐1, NH_2_‐MIL‐101(Fe) and NH_2_‐MIL‐101(Fe)@TpPa‐1. d) FT‐IR spectra of TpPa‐1, NH_2_‐MIL‐101(Fe) and NH_2_‐MIL‐101(Fe)@TpPa‐1. e) N_2_ adsorption isotherm and f) pore size distribution of TpPa‐1, NH_2_‐MIL‐101(Fe) and NH_2_‐MIL‐101(Fe)@TpPa‐1. g) XPS spectrum over NH_2_‐MIL‐101 (Fe)@TpPa‐1 of survey and h) high‐resolution XPS spectrum of N 1s. i) UV−vis absorption spectra of GEM, NRP2, DVDMS, and GDCMN2. j) Fluorescence spectra of NRP‐2 and GDCMN2. k) Zeta Potential of MOF, MOF@COF, GDMC, GDMC‐NH_2_, and GDMCN2.

As shown in Figure S1 (Supporting Information), NH_2_‐MIL‐101(Fe) has an octahedral morphology with a uniform particle size, and the average particle size is ≈250 nm. (Figure S1a,f, Supporting Information). The morphology of GEM‐DVDMS@NH_2_‐MIL‐101 (Fe) did not change significantly, indicating that the GEM and DVDMS loading did not disrupt the MOF structure (Figure S1b, Supporting Information). After surface modification with the COF, GEM‐DVDMS@NH_2_‐MIL‐101 (Fe)@TpPa‐1 COF composite maintained its general morphology (**Figure** **1**a). However, the surface became a slightly rough COF film with a thickness of ≈37 nm (Figure 1a), indicating that the TpPa‐1 COF was successfully coated on NH_2_‐MIL‐101(Fe). Scanning electron microscope (SEM) images of pure TpPa‐1 and NH_2_‐MIL‐101 (Fe)@TpPa‐1 are shown in Figure S1c,d (Supporting Information) respectively. Pure TpPa‐1 exists in the form of nanospheres with an average diameter of 4 µm. Figure S1e (Supporting Information) shows the morphology of NRP2 GEM‐DVDMS@NH_2_‐MIL‐101 (Fe)@TpPa‐1. Different weight ratios of NH_2_‐MIL‐101 (Fe) to TpPa‐1 were prepared. The morphology and Fe EDS analysis are shown in Figure S1 and Table S1 (Supporting Information). From Figure 1b, the water contact angle of NH_2_‐MIL‐101(Fe) is 60.2°, and NH_2_‐MIL‐101(Fe)@TpPa‐1 shows complete water wetting, adsorbing a drop of water immediately, indicating that the hydrophilicity of the composite material was improved with the help of the hydrophilic COF.

The XRD patterns of NH_2_‐MIL‐101 (Fe) [15]and TpPa‐1 ^[^ ^19^ ^]^ (peaks at 4.7° (100 plane) and 27° (001 plane)) were observed for NH_2_‐MIL‐101 (Fe)@TpPa‐1 (Figure 1c), indicating that TpPa‐1 was successfully coated onto the surface of NH_2_‐MIL‐101(Fe). As shown in Figure 1d, the peaks located at 570 cm^−1^ (assigned to Fe─O), 1256 cm^−1^ (assigned to C─N), and 1574 cm^−1^ (attributed to C═ C) in the FT‐IR spectrum of NH_2_‐MIL‐101(Fe)@TpPa‐1 matched well with those of NH_2_‐MIL‐101(Fe) and TpPa‐1.^[^ ^20^ ^]^ The N_2_ adsorption isotherms of the three samples are shown in Figure 1e. NH_2_‐MIL‐101(Fe) exhibits a Type I isotherm, which indicates its typical microporous structure with a surface area of 691 m^2^ g^−1^. The NH_2_‐MIL‐101 (Fe)@TpPa‐1 hybrid material possesses features of Type I and IV isotherms, indicating the coexistence of micropores and mesopores in the MOF@COF hybrid owing to a slight desorption hysteresis between the adsorption and desorption curves. The BET surface area value of MOF@COF (542 m^2^ g^−1^) is lower than that of pure NH_2_‐MIL‐101 (Fe) to varying degrees due to the presence of COF in the hybrid. As expected, pure TpPa‐1 has a minimum BET surface area of (352 m^2^ g^−1^). As shown in Figure 1f, large‐scale peaks located at 1.1 nm and 2.5–4.0 nm were observed in the pore size distribution curve of TpPa‐1, indicating the presence of a mesoporous structure. A peak over 2 nm was also observed in the pore‐size distribution curve of NH_2_‐MIL‐101 (Fe)@TpPa‐1. This result is consistent with the N_2_ adsorption isotherms. The mesoporous structure may be beneficial for the mass transfer and release of drugs. The XPS spectrum of NH_2_‐MIL‐101 (Fe)@TpPa‐1 showed peaks located at binding energies of ≈285, 530, and 711 eV, which are related to C 1s, O 1s, and Fe 2p, respectively (Figure 1g).^[^ ^21^ ^]^ As shown in Figure 1h, the peaks at 400.0 and 399.5 eV were assigned to C─N and C═ N, respectively, indicating the successful synthesis of TpPa‐1 on NH_2_‐MIL‐101 (Fe).^[^ ^22^ ^]^ The characteristic absorption peaks of DVDMS, NRP2, and GEM were distinctly observed in the UV–vis absorption spectrum of GDCMN2 (Figure 1i). As shown in the fluorescence spectra in Figure 1j, the peak position of NRP2 was consistent with that of GDCMN2, indicating that NRP2 was successfully attached to GDCM. The drug‐loading efficiencies (DE) of DVDMS and GEM were 14.4% and 20.3%, respectively. The encapsulation efficiencies (EE) of DVDMS and GEM were 50.6% and 76.6%, respectively (Figure S2, Supporting Information). From Figure 1k, the negative charge of DVDMS and GEM resulted in the zeta potential of GDMC being −24.6 mV, which was significantly lower than the zeta potential of MOF@COF (−9.8 mV). The successful amination of the nanocages led to a potential value of GDMC‐NH_2_ to be +9.22 mV, indicating a positive surface charge. Interestingly, the zeta potential value of GDMCN2 was slightly lower than that of GDMC‐NH_2_ due to the negative charge on the NRP2 mAb. This observation suggests that the NRP2 antibody was successfully loaded onto the GDMC nanocages. To assess the physiological stability of GDMCN2, transmission electron microscopy (TEM) imaging was performed at 24, 72, and 168 h in a standard cellular physiological environment. The observed morphology remained unchanged, indicating the GDMCN2 nanocage's robust stability, with NH2‐MIL‐101(Fe) fully covering the COF shell layer for 168 h, maintaining high crystallinity, and confirming its exceptional stability (Figure S3, Supporting Information).

To assess the release behavior of GDMCN2, we investigated the release profiles of GEM and DVDMS in various PBS buffers at different pH values (pH 7.4, 6.5, and 5.5). The results presented in Figure S4 (Supporting Information) revealed that the cumulative release of GEM and DVDMS at pH 5.5 and 6.5 was notably higher than that at pH 7.4. This observation indicates that the acidic microenvironment present in tumors facilitates the efficient release of GEM and DVDMS from GDMCN2. Additionally, when immersed in PBS at pH 7.4, minimal drug leakage was observed at each time point, suggesting that GDMCN2 possessed excellent drug‐loading stability.

### 2.2 Targeting Ability and Cell Uptake In Vitro

**Figure 2:**
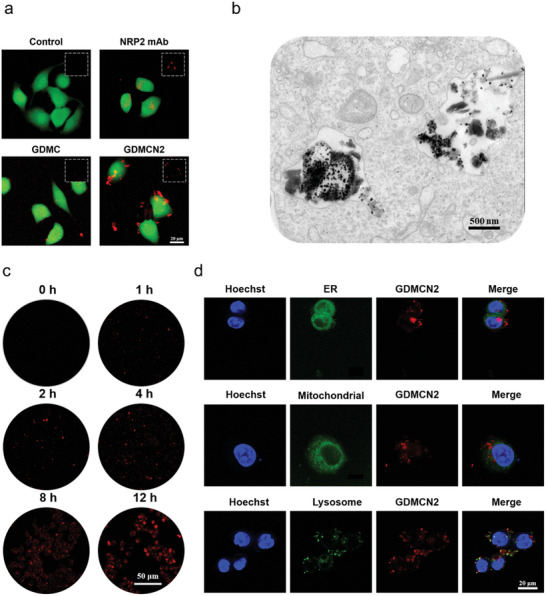
Targeting ability and cell uptake in vitro. a) CLSM images of PANC‐1/GEM cells incubated with RBITC‐labeled NRP2 mAb, RBITC‐labeled GDMC, and RBITC‐labeled GDMCN2 following 0.5 h of incubation. b) Intracellular TEM images of PANC‐1/GEM cells incubated with GDMCN2 following 12 h of incubation. c) CLSM images of PANC‐1/GEM cells following incubation with RBITC‐labeled GDMCN2 for 0, 1, 2, 4, 8, and 12 h. d) CLSM images of PANC‐1/GEM cells after incubation with RBITC‐labeled GDMCN2 for 12 h, followed by ER Green, Mito‐Tracker Green, and LysoTracker Green staining, respectively.

In a previous study, the NRP2 protein was identified as a co‐receptor for multiple growth factors on the cell membranes of pancreatic cancer cells.^[^ ^23^ ^]^ NRP2 mAb specifically binds to NRP2 Confocal laser scanning microscopy (CLSM) was used to examine the endocytosis and targeting abilities of GDMCN2 in PANC‐1/GEM cells. After co‐culturing PANC‐1/GEM cells with RBITC‐labeled NRP2 mAb and RBITC‐labeled GDMCN2 for 0.5 h, the results showed that both proteins were concentrated on the cell membrane. However, no detectable red fluorescence was observed on the cell membrane after incubation with RBITC‐labeled GDMC (**Figure** **2**a). The TEM images of the GDMCN2 and GDMC cell groups revealed a stark distinction. Specifically, a high density of nanocage particles was observed surrounding the cells in the GDMCN2 group, indicating the presence of NRP2. In contrast, the GDMC group showed only a minimal number of particles surrounding the cells. These findings suggest that the NRP2 mAb can target pancreatic cancer cells and that GDMCN2 possesses a targeting ability similar to that of the NRP2 mAb (Figure S5, Supporting Information). To investigate the phagocytosis of the cells on the nanocages, RBITC‐labeled GDMCN2 was incubated with the cells at different times. After 2 h, weak fluorescence was observed in the cytoplasm, which gradually increased with the incubation time (Figure 2c; Figure S6, Supporting Information). Intracellular TEM images showed that GDMCN2 nanocages were prominently present in the cells after incubation for 12 h (Figure 2b). To determine the localization of the organelles after phagocytosis of the nanocages, ER, MitoTracker, and LysoTracker green fluorescent probes were used to detect the ER, mitochondria, and lysosomal organelles, respectively. The results indicated that GDMCN2 was mainly distributed in the membrane system of PANC‐1/GEM cells, with greater distribution in the plasma, nuclear, and organelle membranes, but was not distributed in the nucleus. These results suggest that the nanocages can target the plasma membrane of PANC‐1/GEM cells (Figure 2d). In summary, these findings demonstrated that GDMCN2 can target pancreatic cancer cells and has potential as a drug delivery system. Furthermore, it sheds light on the localization of nanocages after their incorporation into cells, which could facilitate the development of more effective and targeted drug delivery systems for the treatment of pancreatic cancer.

### 2.3 In Vitro SDT Assessment

**Figure 3:**
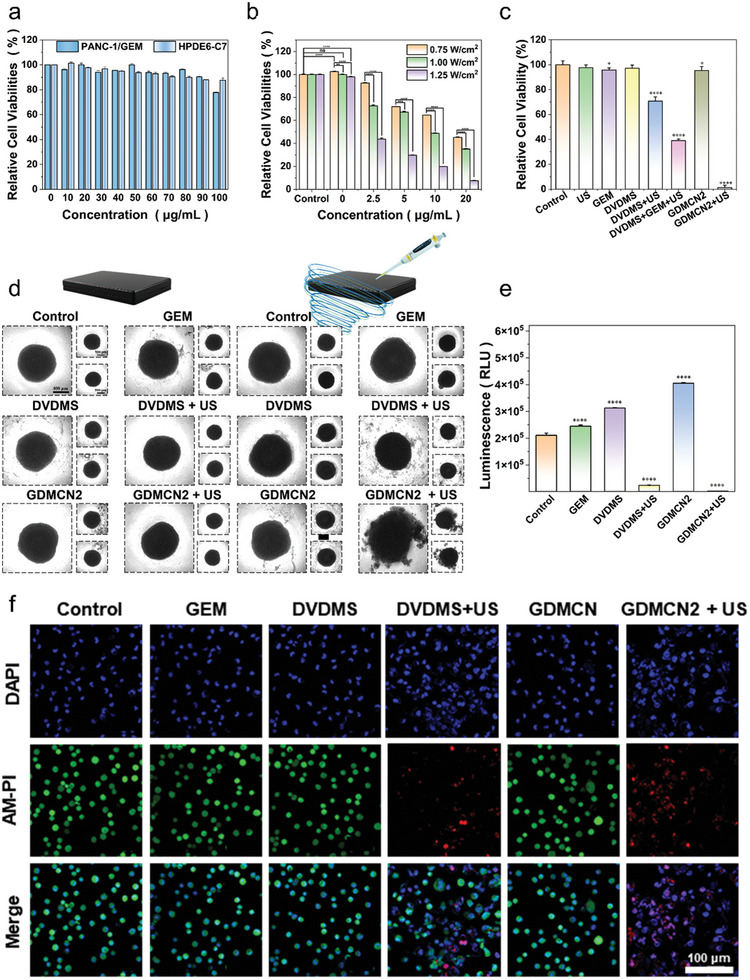
In vitro safety and therapeutic effect of GDMCN2 under sonodynamic therapy. a) In vitro safety at different concentrations of GDMC in PANC‐1/GEM. b) Relative viability of PANC‐1/GEM cells with ultrasonic irradiation under different power and concentrations (Irradiation time: 2 min). c) Relative viability of PANC‐1/GEM cells with different treatments. d) morphology in a 3D tumor spheroid after different treatments. e) ATP content in a 3D tumor spheroid after different treatments. f) CLSM images of PANC‐1/GEM stained with calcein‐AM and PI followed with different treatments.

To expand their biological applications, the intracellular cytotoxicity of GDMCN2 nanocages was assessed. PANC‐1/GEM and HPDE6‐C7 cells were incubated with various concentrations of GDMCN2 nanocages and cell viability was determined using a cell viability assay reagent (CCK‐8). Even at a concentration of 100 µg mL^−1^, the survival rate of PANC‐1/GEM cells was 77%, and that of HPDE6‐C7 cells was 87%, demonstrating that GDMCN2 nanocages have outstanding biocompatibility (**Figure** **3**a). Subsequently, the effect of the GDMCN2 nanocages on cell death under US irradiation was explored. When the treatment time was 2 min, US at power levels of 0.75 W/cm^2^, 1.0 MHz, and 1 W/cm^2^, 1.0 MHz had no significant impact on the cell viability. However, at 1.25 W/cm^2^, 1.0 MHz power, it led to mechanical damage, resulting in a cell viability of 97% compared to the control group. Moreover, the degree of cell damage showed a notable dependence on both GDMCN2 nanocage concentration and ultrasonic power. Based on the results presented in Figure 3b and these findings, we confidently determined that using 1 W/cm ^2^, 1.0 MHz power for 2 min of treatment with US alone does not cause detrimental effects on cell viability. Thus, subsequent experiments followed the parameters of 1 W/cm ^2^, 1.0 MHz power, and 2‐min duration of sonication to preserve cell integrity and viability during treatment.

To investigate the therapeutic effects of different treatment groups on PANC‐1/GEM cells, the cells were divided into seven groups: control, GEM, DVDMS, DVDMS combined with US irradiation (DVDMS+US; 1 W/cm^−2^, 2 min, 1.0 MHz), DVDMS combined with GEM and US irradiation (DVDMS+GEM+US; 1 W/cm^−2^, 2 min, 1.0 MHz), GDMCN2, and GDMCN2 combined with US irradiation (GDMCN2+US; 1 W/cm^−2^, 2 min, 1.0 MHz). Nanocage concentration is 20 µg mL^−1^ (equivalent to DVDMS 2.88 µg mL^−1^, GEM 4 µg mL^−1^). Based on the results presented in Figure 3c, compared to the control group, the cell viability of the GEM and US‐only groups were 95.7% and 97.6%, respectively; and that of the DVDMS+US group and DVDMS+GEM+US group were 70%, and 40%, respectively. According to the literature, DVDMS, as a sound sensitizer, may generate ROS in cells after ultrasonic excitation, making it easier for GEM to combine with cellular DNA and play a role. However, cell viability in the GDMCN2+US group was only 1.33%, which may be due to the targeting of NRP2, which increased the phagocytosis of GDMCN2, completely destroying the homeostasis of the cells and jointly exerting antitumor effects with GEM.^[^ ^24^ ^]^

Annexin V/FITC was detected using flow cytometry, and the results were consistent with those of the CCK‐8 assay (Figure S7, Supporting Information). Live/dead cell differentiation was observed using CLSM, and the GDMCN2+US group induced the most significant cell death, as indicated by the presence of red fluorescence (Figure 3f; Figure S8, Supporting Information). We minimized the potential toxicity of DAPI on live cells, and the concentration and exposure time were carefully controlled to ensure acceptable effects on cell viability.^[^ ^25^ ^]^

To further explore the therapeutic effect of GDMCN2 nanocages in tumor tissues, PANC‐1/GEM 3D cell spheres were constructed in vitro. After US irradiation, the outermost cells of the DVDMS+US group were scattered, whereas the spheroids in the GDMCN2‐bound US group were destroyed and lost their original spheroid morphology (Figure 3d). Using the CellTiter‐Lum Luminescent 3D Cell Viability Detection Kit, the overall activity of the 3D cultured cells was evaluated, and the results demonstrated that GDMCN2 nanocages significantly decreased the viability of tumor cell spheroids following US irradiation, with an average relative light unit (RLU) of 2316 compared to 24089 for the DVDMS+US group, and 210657 for the control group. These results indicated that GDMCN2 nanocages may function as potent sonosensitizers for SDT in cancer therapy (Figure 3e).

### 2.4 In Vitro Oxidative Stress and Antitumor Mechanisms

**Figure 4:**
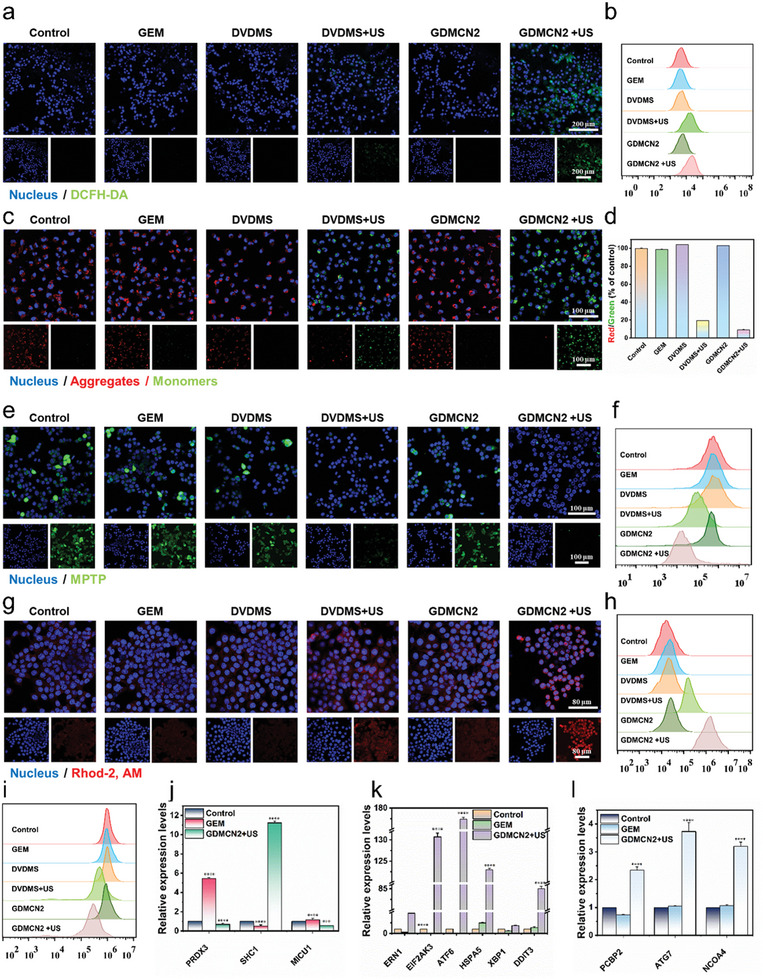
In vitro oxidative stress and antitumor mechanisms. a and b) Cellular CLSM images and flow cytometric analyses of DCFH‐DA‐stained PANC‐1/GEM cells exposed to different treatments. c and d) JC‐1 stained PANC‐1/GEM cells as seen by CLSM with the appropriate red‐to‐green fluorescence intensity ratio. e,f) PANC‐1/GEM cells stained with MPTP and analyzed using a flow cytometer in a CLSM. g and h) PANC‐1/GEM cells stained with Rhod‐2, AM and examined by a CLSM and a flow cytometer for their responses to different treatments. i) MitoTracker staining of PANC‐1/GEM cells under various conditions and analyzed by flow cytometry. j–l) Intracellular endoplasmic reticulum stress, mitochondrial calcium damage, autophagy‐related RT‐qPCR relative expression level under different treatments.

The remarkable cell‐killing effect of GDMCN2 prompted us to investigate its mechanism of action under various conditions. To measure intracellular ROS, we employed 2,7‐dichlorodiacetic acid fluorescein (DCFH‐DA), a widely used ROS indicator that can generate green fluorescent DCF upon oxidation of non‐fluorescent DCFH. As expected, we observed no green fluorescence in the control, GEM, DVDMS, and GDMCN2 treatment groups, with only weak fluorescence observed in the DVDMS+US group, indicating that DVDMS has a sonodynamic effect and can generate ROS when exposed to US. Notably, the GDMCN2+US group exhibited a significantly higher green fluorescence, as shown in **Figure** **4**a and Figure S9 (Supporting Information). These results were corroborated by flow cytometry data, which showed that tumor cells exhibited the strongest fluorescence intensity under GDMCN2+US treatment compared with the other groups, as shown in Figure 4b. Mitochondria, the energy centers of cells, are highly susceptible to ROS because of their membrane structure and internal environment.^[^ ^26^ ^]^ Therefore, we stained the mitochondria of PANC‐1/GEM cells with tetraethylbenzimidazolyl‐carbocyanine iodide (JC‐1) to determine the mitochondrial membrane potential.^[^ ^27^ ^]^ Mitochondrial membrane potential depolarization is represented by red fluorescence due to the formation of JC‐1 aggregates in mitochondria and by green fluorescence due to the presence of JC‐1 monomers that are not linked with the mitochondrial inner membrane and hence remain in the cytoplasm. As depicted in Figure 4c, compared to other treatment groups, the green fluorescence of PANC‐1/GEM cells significantly increased after irradiation with GDMCN2 and US (1 W cm^−2^, 2 min, 1.0 MHz), indicating obvious depolarization of the mitochondrial membrane potential. Similar results were obtained when the ratio of red to green fluorescence was calculated, indicating that mitochondrial depolarization occurred when GDMCN2 was introduced into the cells and US irradiation was applied, as shown in Figure 4d. Based on the results shown in Figure 3c and Figure S10 (Supporting Information), we addressed the concerns related to cell damage and reduced mitochondrial membrane potential resulting from US alone at a power level of 1 W cm^−2^. These findings confirm that US exposure at this intensity does not exert detrimental effects on cell viability or mitochondrial activity.

The mitochondrial permeability transition pore (MPTP) is a highly regulated channel with various ion channel activities.^[^ ^28^ ^]^ A reduced mitochondrial membrane potential can result in the opening of MPTP, leading to abnormal mitochondrial function. An MPTP detection kit was used to determine the degree of influence of the different treatments on the MPTP of PANC‐1/GEM cells. The results showed that the control, GEM, DVDMS, and GDMCN2 groups exhibited higher mitochondrial membrane potentials, causing only cytoplasmic fluorescence to be quenched by CoCl_2_ whereas mitochondria displayed strong green fluorescence (Figure 4e). A comparison between the DVDMS+US group and the GDMCN2+US group revealed that the latter group had a lower mitochondrial potential. CoCl_2_ completely quenched the fluorescence in the mitochondria, indicating severe damage to the mitochondrial function of GDMCN2 after US irradiation and the complete activation of MPTP. These results were confirmed by flow cytometry (Figure 4f).

It has been reported that an increase in Ca^2+^ within the mitochondria can result in MPTP opening, leading to further mitochondrial membrane potential decline and dysfunction.^[^ ^29^ ^]^ To investigate the Ca^2+^ level in the mitochondria after MPTP opening, we employed the mitochondria‐specific Ca^2+^ fluorescent probe Rhod‐2, which is specifically localized to the mitochondria, with fluorescence intensity positively correlated with Ca^2+^ concentration.^[^ ^30^ ^]^ As shown in Figure 4g, compared to the control group, the DVDMS+US group exhibited stronger red fluorescence, whereas the GDMCN2+US group showed a further increase in fluorescence intensity. Flow cytometry results confirmed the same fluorescence intensity as that observed in the CLSM images (Figure 4h). These findings suggest that following US irradiation, GDMCN2 interacts with the mitochondria, leading to Ca^2+^ overload and exacerbation of mitochondrial damage.

We subsequently used the mitochondrial probe MitoTracker Green to detect changes in mitochondrial mass across the different treatment groups.^[^ ^31^ ^]^ The results showed that, compared with other groups, the fluorescence intensity in the GDMCN2+US irradiation group was significantly reduced, indicating more severe mitochondrial damage, and decreased mitochondrial quality (Figure 4i). Real‐time fluorescent quantitative PCR (RT‐qPCR) analysis was performed to further investigate the impact of mitochondrial Ca^2+^ excess on damage‐related gene expression. Given the resistance of PANC‐1/GEM to GEM, we compared the GDMCN2+US and GEM groups. The results revealed that following US irradiation, GDMCN2 disrupted mitochondrial peroxisome deactivating enzyme (PRDX3) and mitochondrial Ca^2+^ imbalance regulatory protein (MICU1) function, leading to significant inhibition of the RNA expression of these genes while activating the pigment enzyme P66Shc‐related gene SHC1 (Figure 4j). Overall, these results suggest that after GDMCN2 enters the cells and undergoes US irradiation, a large amount of ROS is generated, leading to an imbalance in mitochondrial Ca^2+^ and subsequent mitochondrial damage, and dysfunction, affecting related genes. These findings provide important insights into the mechanisms underlying the effects of GDMCN2 and US on cellular function and may have significant therapeutic implications.

ER is essential for protein synthesis and folding within cells.^[^ ^32^ ^]^ An increase in Ca^2+^ in the mitochondria leads to the opening of the MPTP, resulting in the release of Ca^2+^ into the cytoplasm. This calcium‐ER stress coupling is responsible for ER dysfunction and ER stress.^[^ ^33^ ^]^ The RT‐qPCR results in Figure 4k reveal that GDMCN2 nanocages induced mitochondrial damage following US irradiation, activating ER stress sensors such as IRE1, PERK, and ATF6. Subsequently, the downstream genes HSPA5, XBP1, and DDIT3 were significantly activated. XBP1s play a crucial role in cellular response to ER stress. Upon induction of ER stress, the precursor mRNA of XBP1 is activated and subsequently cleaved to form the active XBP1s protein. This protein is essential for maintaining cellular homeostasis and for facilitating cellular adaptation to environmental changes. Figure S11a (Supporting Information) illustrates the increased expression of XBP1s following DVDMS+US treatment. Interestingly, US irradiation led to a more pronounced increase in the expression of GDMCN2, indicating its potential involvement in the stress response pathway. ATF6 and ERN1 are key molecules involved in ER stress pathway. Under stressful conditions, they are activated to enhance cellular stress resistance by regulating the transcription of specific genes, facilitating proper protein folding, and promoting cellular homeostasis. Supporting these observations, Figure S12 (Supporting Information) shows strong green fluorescence in the GDMCN2+US group in cellular immunofluorescence, indicative of significant activation of ATF6 and ERN1. Considering the results depicted in Figure 4k along with these findings, it is evident that GDMCN2+US treatment effectively induces ER stress in cells.^[^ ^34^ ^]^

The LC3 protein plays a pivotal role in cellular autophagy. Upon the initiation of autophagy, LC3 protein binds to the autophagic vacuole membrane to form LC3‐II. Thus, LC3 is a widely utilized marker for studying and assessing the activity and functionality of autophagy. Figure S9a (Supporting Information) shows the significant increase in the expression of LC3‐II in the GDMCN2+US group. Additionally, Figure S13 (Supporting Information) shows the successful observation of intracellular autophagosomes in the GDMCN2+US group. Consequently, in conjunction with the results shown in Figure 4l, it is reasonable to propose that GDMCN2+US effectively induces autophagy in cells.^[^ ^35^ ^]^

These results prove that GDMCN2 nanocages can induce ER stress and autophagy in PANC‐1/GEM cells under US irradiation. The interplay between ER stress and DNA damage is complex, and existing literature highlights that ER stress can lead to ER dysfunction, resulting in the accumulation of unfolded or misfolded proteins and subsequent oxidative damage to DNA. Moreover, under ER stress, cellular resources and energy may be preferentially allocated to cope with ER stress, leading to reduced priority of DNA damage repair. Consequently, this imbalance can result in the accumulation of DNA damage and a decrease in the ability of the cells to effectively repair it.

### 2.5 In Vitro DNA Damage for SDT

GDMCN2 can substantially destroy the mitochondria of GEM‐resistant PANC‐1/GEM pancreatic cancer cells and promote ER stress and autophagy. However, ER stress and elevated Ca^2+^ concentration may result in oxidative stress and DNA damage. In this study, two methods were used to detect DNA damage in cells treated with GDMCN2 and irradiated with US.

**Figure 5:**
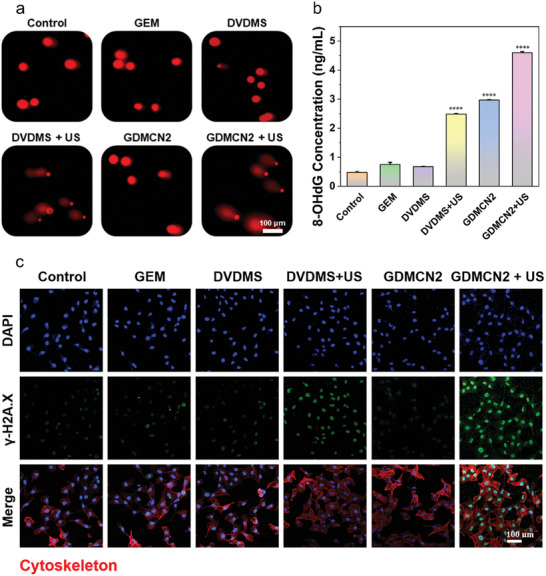
In vitro DNA damage for sonodynamic therapy. a) Electrophoresis of single‐cell gels of PANC‐1/GEM cells subjected to various interventions. b) ELISA analysis of 8‐OHdG concentrations in PANC‐1/GEM cells following different treatments. c) the immunofluorescence of the γ‐H2A.X protein in PANC‐1/GEM cells following various therapies.

Single‐cell gel electrophoresis was used to analyze how GDMCN2 damage alters the physical and chemical properties of DNA.^[^ ^36^ ^]^ The tail length and DNA content of comets reflect the degree of DNA damage in cells.^[^ ^37^ ^]^ The results showed that DNA in the control, GEM, DVDMS, and GDMCN2 treatment groups remained undamaged, whereas DVDMS+US treatment caused DNA fragmentation and the formation of a comet tail (**Figure** **5**a). Furthermore, treatment with GDMCN2+US resulted in more severe DNA fragmentation, longer migration distance, longer comet tail, and enhanced fluorescence intensity. Next, the formation of products after DNA damage was assessed using 8‐hydroxy‐2′‐deoxyguanosine (8‐OHdG) as a biomarker.^[^ ^38^ ^]^ The results showed that 8‐OHdG levels in the control group were 0.48 ng mL^−1^, whereas those in the DVDMS+US group were 2.48 ng mL^−1^. Interestingly, the levels in the GDMCN2+US group were significantly higher at 4.6 ng mL^−1^ (Figure 5b). Finally, the intracellular DNA double‐strand break marker, γ‐H2A.X was examined.^[^ ^39^ ^]^ Cellular immunofluorescence results showed weak green fluorescence in the control, GEM, DVDMS, and GDMCN2 treatment groups, whereas fluorescence intensity in the DVDMS+US group was enhanced. In contrast, the GDMCN2+US group showed the strongest green fluorescence, indicating many DNA double‐strand breaks and severe damage (Figure 5c and Figure S14, Supporting Information). Figure S15 (Supporting Information) shows that the expression of DNA repair genes associated with drug resistance is substantially downregulated in pancreatic cancer. Our results demonstrated that GDMCN2 nanocages stimulated by US irradiation can severely damage DNA in cells and increase DNA damage product formation. This lays the foundation for GEM to enter the DNA of the drug‐resistant PANC‐1/GEM cells and exert its therapeutic effects.

### 2.6 In Vitro Cell Proliferation Ability

**Figure 6:**
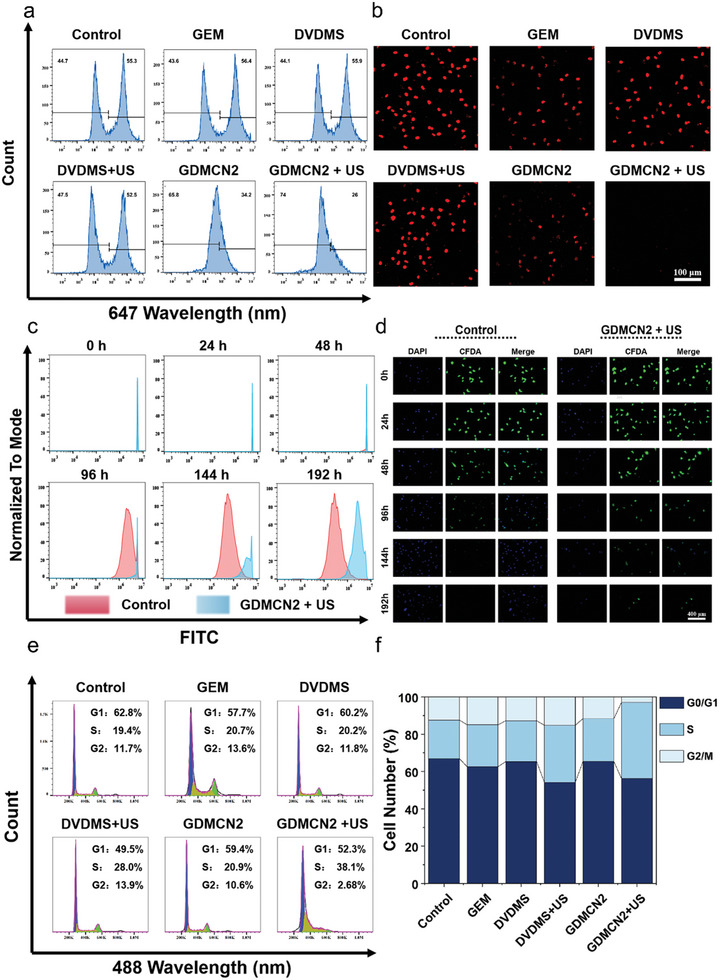
In vitro cell proliferation ability. a) The percentage of EdU‐positive cells in treated PANC‐1/GEM cells was determined by flow cytometry. b) CLSM images of PANC‐1/GEM cells stained with EdU under different treatments. c and d) The proliferation ability of CFSE‐labeled PANC/GEM cells was analyzed by flow cytometry and CLSM images, respectively. e) Using flow cytometry, the cell cycle of PANC‐1/GEM cells subjected to various regimens was determined. f) Analyzing the statistical distribution of PANC‐1/GEM cell proliferation by therapy.

Regarding drug resistance in pancreatic cancer, previous studies have confirmed that GDMCN2 overcomes drug resistance by damaging the DNA of PANC‐1/GEM cells. GEM can interfere with DNA synthesis and DNA chain extension in tumor cells, thereby inhibiting the proliferation and growth of cancer cells.^[^ ^40^ ^]^ Next, the ability of PANC‐1/GEM cells to proliferate after different treatments was determined. DNA synthesis and proliferative activity of the cells were measured using the thymidine analog 5‐ethynyl‐2′‐deoxyuridine (EdU) to determine the effect of the regimens.^[^ ^41^ ^]^ The percentage of EdU‐positive cells across study groups was determined using flow cytometry. The results showed that the fraction of EdU‐positive cells was 26% when GDMCN2 was coupled with US, and that EdU‐labeled DNA synthesis and cell proliferation were severely suppressed. EdU‐positive cells were more common among drug‐resistant PANC‐1/GEM cells in the GEM and control groups (55.3% and 56.4%, respectively). As GEM was encapsulated within the nanocage, increasing its chance of entering the cells, cell proliferation was inhibited in the GDMCN2 group, with only 34.2% positive cells(**Figure** **6**a). Consistent with the flow cytometry results, CLSM demonstrated that the fluorescence intensity of the GDMCN2+US group was negligible, whereas the control and GEM groups exhibited higher intensities (Figure 6b). Furthermore, live cell proliferation was tracked using CFDA SE, a fluorescent dye that allows the tracking of cell division.^[^ ^42^ ^]^ Fluorescence was observed using CLSM and the fluorescence intensity of CFSE‐labeled PANC‐1/GEM cells was evaluated using flow cytometry at various time points. As shown in Figure 6c,d, we assessed the cell proliferation ability of the GDMCN2+US and control groups at different time points using flow cytometry and fluorescence intensity. The results revealed that cells treated with GDMCN2+US exhibited a slower decrease in fluorescence intensity than control cells. Notably, after 144 h of observation, the fluorescence intensity in the control group nearly disappeared, whereas the GDMCN2+US group retained a weak fluorescence signal. This finding strongly suggests that the proliferative ability of PANC‐1/GEM cells was significantly impaired following phagocytosis of GDMCN2 nanocages and US irradiation. This result is consistent with the EdU findings. The effects of the different treatments on the cell division cycle of PANC‐1/GEM cells were determined using propidium iodide (PI), a fluorescent dye used for double‐stranded DNA. The percentage of cells in each cell cycle phase was also determined. The percentage of cells in the G1 and S phases increased to 90.4% compared to the control group, while the percentage of cells in the G2/M phase decreased to just 2.68% in the GDMCN2+US treatment group. These results imply that GEM can prevent PANC‐1/GEM cells from dividing and maintain them in the G1 and S phases of the cell cycle (Figure 6e and f). Overall, the results of this study showed that following exposure to US irradiation, GDMCN2 damaged the DNA of PANC‐1/GEM cells, allowing GEM to suppress tumor cell proliferation by interfering with DNA synthesis and DNA chain extension. Moreover, we clarified the rationale behind the lack of significant effects on cell proliferation in the DVDMS+US group, which was further corroborated by RT‐qPCR analysis of relevant genes (Figure S16, Supporting Information).

### 2.7 GDMCN2 is Activated by US to Promote Cell Ferroptosis

**Figure 7:**
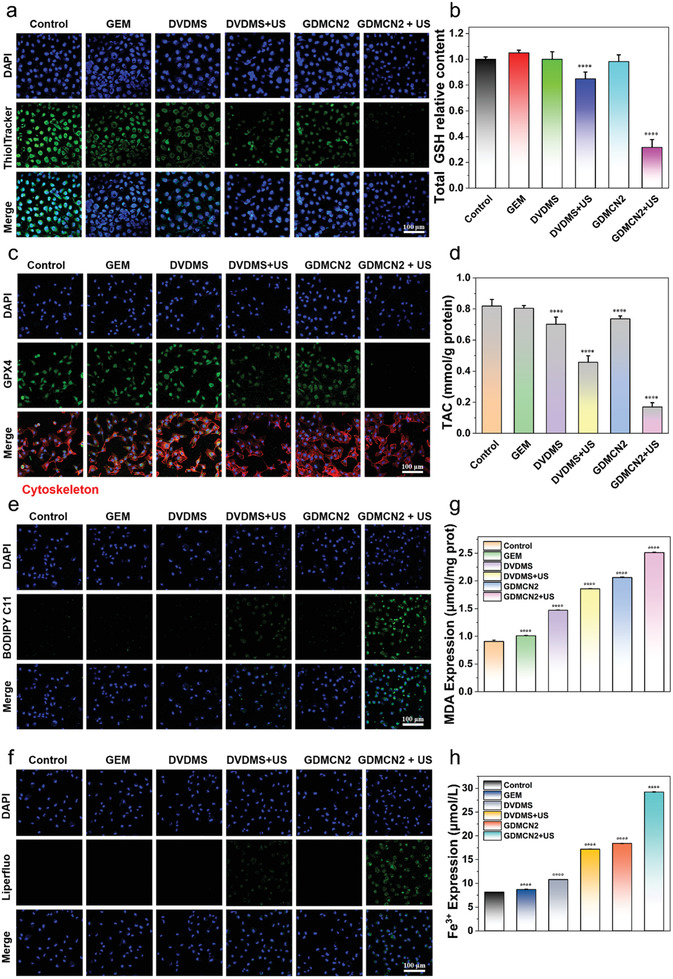
GDMCN2 is activated by ultrasound to promote cell ferroptosis. a) CLSM images of intracellular GSH in PANC‐1/GEM cells with different treatments. ThiolTracker Violet discolored the GSH level (green). b) The total GSH level in cells under the different groups. c) CLSM image of GPX4 protein immunofluorescence in PANC‐1/GEM cells treated in the different groups. d) Assessment of total antioxidant capacity in PANC‐1/GEM cells treated in the different groups. e,f) CLSM images of PANC‐1/GEM cells that were stained with BODIPY and Liperfluo in the different groups, respectively. g,h) MDA expression and Fe^3+^ content in PANC‐1/GEM cells treated in the different groups.

Previous research indicated that under US irradiation, GDMCN2 nanocages cause mitochondrial damage, leading to ER stress and subsequent DNA damage. This process allows GEM to overcome drug resistance and enter the cell DNA, thereby inhibiting cell proliferation. However, drug resistance can also arise from the enhanced antioxidant capacity of cells, resulting in increased cellular DNA repair capacity.^[^ ^43^ ^]^ Therefore, we investigated the antioxidant capacity and mode of death of PANC‐1/GEM cells. Reduced glutathione (GSH) is a representative intracellular antioxidant, and its content is positively correlated with the intracellular antioxidant system.^[^ ^44^ ^]^ As shown in **Figure** **7**a and Figure S17 (Supporting Information), the detection of intracellular GSH content using ThiolTracker Violet revealed that cells in the control group exhibited robust green fluorescence due to the absence of interventions. Conversely, the GEM, DVDMS, and GDMCN2 groups exhibited weaker fluorescence intensities owing to the consumption of GSH as part of the cellular self‐antioxidation process. Both GSH and oxidized glutathione (GSSG) regulate the cellular redox environment. Subsequently, we determined the total intracellular GSH levels and discovered that the GDMCN2+US group had a GSH content that was only 0.31 times that of the control group, whereas the DVDMS+US group had a GSH content that was 0.84 times that of the control group (Figure 7b). These results are consistent with the CLSM graph. We also quantitatively analyzed the total antioxidant capacity of cells after GSH reduction using the ABTS method.^[^ ^45^ ^]^ The antioxidant capability of the drug‐resistant PANC‐1/GEM cells was destroyed, as shown by the drastic decrease in the control group (0.8188 mmol g^−1^ protein) as compared to the GDMCN2+US group (0.1698 mmol g^−1^ protein) (Figure 7d). GPX4 is a crucial enzyme that converts lipid peroxides into alcohols that are harmless to cells.^[^ ^46^ ^]^ GPX4 protects the cells from lipid peroxide damage and maintains cellular homeostasis. GSH acts as a cofactor for GPX4 and its activity requires the participation of GSH, which acts as a reducing agent to maintain its activity. Western blotting and immunofluorescence techniques were employed to examine the alterations in GPX4 levels in cells following different treatments. Our findings revealed that upon subjecting GDMCN2 to US irradiation, there was a remarkable reduction in the GPX4 protein content in PANC‐1/GEM cells (Figure 7c, Figures S18 and S19, Supporting Information). Lipid peroxide is a potent oxidizing substance that severely damages cell membranes and other cellular components, leading to ferroptosis.^[^ ^47^ ^]^ In conjunction with Figure 4l and Figure S13 (Supporting Information), which show a significant increase in autophagic lysosomes in cells, we became interested in whether GDMCN2 undergoes ferroptosis after US irradiation after entering the cells.

We examined the changes in intracellular lipids, lipid peroxides, and the metabolite malondialdehyde (MDA) of lipid peroxides in the different treatment groups. The intracellular lipid probe BODIPY C11 (Figure 7e and Figure S20, Supporting Information) and the lipid peroxide probe LiperFluo (Figure 7f and Figure S21, Supporting Information) revealed that the fluorescence intensity of the GDMCN2+US irradiation group was significantly higher than that of the other treatment groups. The quantitative MDA content in the control group was 0.90 µmol mg^−1^ protein, whereas that in the GDMCN2+US group was 2.51 µmol mg^−1^ protein (Figure 7g). These findings suggest that an abundance of intracellular lipid peroxides accumulate because of GSH and GPX4 inactivation. These results indicate that the accumulation of intracellular lipid peroxides increased owing to the inactivation of GSH and GPX4.

In summary, our findings suggest that inactivation of GSH and GPX4 leads to the accumulation of lipid peroxides in cells. Furthermore, US stimulation of GDMCN2 triggers severe damage to cellular organelles, inducing autophagy through the activation of calcium and iron ions. Finally, autophagy‐dependent ferroptosis occurs in these cells. These observations provide new insights into the potential mechanisms underlying ferroptosis induction and highlight the importance of understanding the role of GDMCN2 in US‐mediated ferroptosis.

### 2.8 In Vivo Safety and Distributions of GDMCN2 Nanocage

Prior to tail vein injection, the biocompatibility of GDMCN2 was assessed in vivo. The GDMCN2 solution at a concentration of 40 µg mL^−1^ exhibited no significant hemolysis, as shown in Figure S22 (Supporting Information). These findings confirm the favorable biocompatibility of GDMCN2 with blood, establishing its potential suitability for in vivo applications with a promising safety profile.

**Figure 8:**
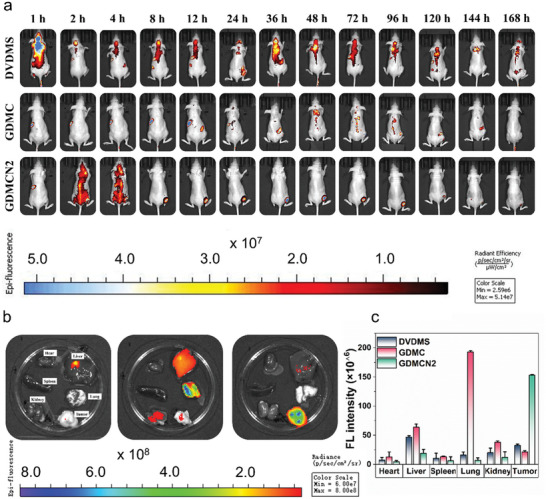
In vivo distributions of GDMCN2 nanocage at different times. a and b) Nude mice with tumors were imaged intravenously with different formulations and were also imaged ex vivo including critical organs. c) Quantitative analysis of ex vivo fluorescence images collected at different time intervals.

We evaluated their distribution and targeted their abilities in vivo. Subsequently, PANC‐1/GEM cells were injected subcutaneously into BALB/c nude mice, and when the tumor volume reached 100 mm^3^, the mice were randomly divided into three groups based on their DVDMS content: DVDMS, cy5.5‐labeled GDMC, and cy5.5‐labeled GDMCN2 groups. The lack of targeting ability and short blood retention duration in the DVDMS group led to a poor fluorescent signal at the tumor site, as seen in the imaging data; instead, the group concentrated primarily on the liver after 1 h. Instead, primary tumor accumulation was observed within 2 h in the GDMCN2 groups, with GDMCN2 exhibiting good tumor‐targeting capacity and sustaining a relatively strong fluorescent signal even 168 h post‐injection. Collectively, these data show that GDMCN2 is highly effective in targeting and retaining tumors with few off‐target effects (**Figure** **8**a). Ex vivo imaging of tumors, major tissues, and organs was also carried out, with high fluorescent signals identified in tumor tissue in the GDMCN2 group, and weak signals detected in the lungs and liver, but essentially no signal in other tissues and organs (Figure 8b,c). These results show that ex vivo organ imaging agreed with in vivo imaging, providing further evidence of GDMCN2's effective targeting capacity.

### 2.9 In Vivo Antitumor Efficiency of GDMCN2 for SDT

**Figure 9:**
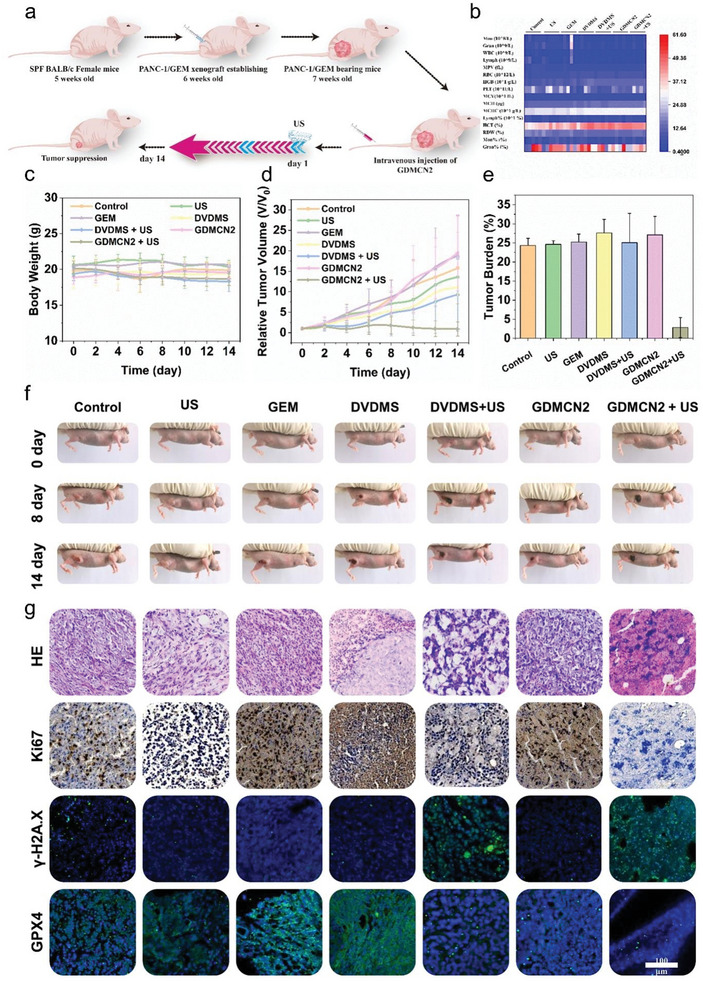
In vivo antitumor efficiency of GDMCN2 for sonodynamic therapy. a) Schematic depiction of the treatment plan for sonodynamic therapy in the PANC‐1/GEM tumor model mediated by GMCMN2. b) Mice hematological index on the 14th day following different treatments. c) Mice body weight versus treatment time. d) Tumor burden of all groups. e) Relative tumor volume (*v*/*v*_0_) versus treatment time. f) On the 0th, 8th, and 14th day, representative images were taken of mice from the different groups. g) Images demonstrate H&E staining, Ki‐67 immunohistochemistry, γ‐H2A.X, and GPX4 immunofluorescence of tumors collected on day 14 of different therapies.

We used a PANC‐1/GEM tumor‐bearing mice model to investigate the tumor‐suppressive effect of SDT in vivo, inspired by the encouraging findings of GDMCN2 under sonodynamic in vitro. Mice with tumors were randomly assigned to one of the following six groups: PBS, GEM, DVDMS, DVDMS+US (1.0 MHz, 1.25 W cm^−2^, 6 min), GDMCN2, or GDMCN2+US (1.25 W cm^−2^). On day 1, mice were injected intravenously with GDMCN2 for 12 h and then treated using the matching procedure (**Figure** **9**a). Every other day, the body weight and tumor girth were recorded. Within two weeks, no change in body weight was observed in any of the treatment groups (Figure 9c; Table S2, Supporting Information), and the hematological indicators of the mice in the GDMCN2+US group were within the normal range, indicating the excellent therapeutic safety of GDMCN2 (Figure 9b; Table S3, Supporting Information). Tumor volume measurements revealed that the PBS, DVDMS, and GDMCN2 groups had minimal treatment effects. In contrast, DVDMS+US and GDMCN2+US groups showed significant antitumor effects (Figure 9d). Notably, due to the emergence of drug resistance of PANC‐1/GEM, treatment with the same concentration of GEM did not inhibit tumor growth. Representative digital images (Figure 9f and Figure S23, Supporting Information) of mice showing tumor weight, tumor burden (Figure 9e), and ex vivo tumor tissues (Figure S24, Supporting Information) visually reflect the excellent antitumor therapeutic effects of GDMCN2 under SDT conditions. These findings suggest that GDMCN2 has the potential to be an effective therapeutic agent for cancer.

To further investigate the therapeutic effect of GDMCN2, we performed immunohistochemical staining of the cell proliferation marker Ki‐67, immunofluorescence staining of γ‐H2A.X and GPX4, and hematoxylin and eosin (HE) staining on differently treated tumor sections (Figure 9g). In Ki‐67 immunohistochemistry, the proportion of Ki‐67‐positive proliferating cells in the GDMCN2+US group was considerably lower than those in other groups. Immunofluorescence staining of γ‐H2A.X and GPX4 showed that after treatment with GDMCN2+US, the fluorescence intensity of γ‐H2A.X increased significantly, whereas GPX4 fluorescence intensity was significantly weaker than those in other treatment groups. These results indicate severe DNA damage and ferroptosis in these cells. Figure S25 (Supporting Information) demonstrates the evaluation of tumor tissue mitochondrial function using the marker Translocase of Outer Mitochondrial Membrane 20 (TOM20). The control group showed intact mitochondrial morphology, while the DVDMS+US group displayed damage and non‐specific staining. In the GDMCN2+US group, TOM20 content decreased alongside nuclear rupture. US alone had minimal impact on tumor tissues. HE staining results showed more severe tumor cell death in the tumor sections of the GDMCN2+US treatment group.

Furthermore, HE images of key tissues and organs, such as the heart, liver, spleen, lung, kidney, skin, muscle, and brain, were analyzed in each group to evaluate the biosafety and biocompatibility of GDMCN2 during US treatment (Figure S26, Supporting Information). The results showed no evidence of pathological changes or organ damage, indicating that GDMCN2+US treatment exhibits excellent tissue compatibility and therapeutic biosafety. These results demonstrate that GDMCN2 nanocages subjected to SDT can induce DNA damage in drug‐resistant tumor tissues, resulting in cell ferroptosis and providing an effective strategy for the treatment of drug‐resistant tumors. This approach has great potential for clinical translation as a safe and effective treatment strategy for drug‐resistant tumors.

## 3 Conclusion

In conclusion, the development of drug‐resistant tumors is a major challenge in cancer therapy, and the search for effective strategies to overcome this challenge is ongoing. In this study, we designed and synthesized a novel GDMCN2 nanocage with pancreatic cancer‐specific targeting, which showed remarkable efficacy in eliminating GEM‐resistant pancreatic cancer cells. Our results revealed that sonodynamic stimulation generated large amounts of ROS, leading to mitochondrial and DNA damage, ER stress induction, and ultimately triggering autophagy‐dependent ferroptosis. Importantly, the GDMCN2 nanocage efficiently targeted and delivered the sonosensitizer and GEM to the tumor cells, thereby increasing the effectiveness of SDT and overcoming drug resistance. Our findings highlight the potential of GDMCN2 nanocages as an effective therapeutic strategy for the clinical elimination of GEM‐resistant pancreatic cancer. Further research is required to optimize the delivery system and evaluate the safety and efficacy of this therapy in preclinical and clinical trials. In summary, our study opens the door for the development of more effective cancer treatments by providing novel insights and strategies for combating drug resistance in pancreatic cancer.

## 4 Experimental Section

### Materials

Terephthalic acid (99%), ferric chloride hexahydrate (98%), 2,4,6‐triformylphloroglucinol (Tp) (97%), mesitylene (98%), 1,4‐dioxane (99%), and p‐phenylenediamine (Pa‐1) (98%) were purchased from Sigma‐Aldrich. Hoechst 33342, ThiolTrace Violet, the 2,7‐dichlorofluorescein diacetate (DCFH‐DA), and liperfluo were purchased from DOJINDO. BODIPYTM 581/591 C11, Tetraethylbenzimi‐dazolylcarbocyanine iodide (JC‐1), and Gemcitabine (GEM) were purchased from MCE. Dulbecco's modified Eagle's medium (DMEM) medium was purchased from Thermo Fisher Scientific. The Cell Counting Kit‐8 (CCK‐8), CFDA SE Cell Proliferation and Cell Tracking Kit, Cell Cycle and Apoptosis Analysis Kit, Calcein‐AM/PI Double Stain Kit, Rhod‐2 AM, ER‐Tracker Green, MitoTracker Green FM, and LysoTracker Green DND‐26 were purchased from Yeasen, China. GSH and GSSG Assay Kits, Total Antioxidant Capacity Assay Kit using ABTS technique, CellTiter‐Lumi Luminescent 3D Cell Viability Assay Kit, BeyoClic EdU Cell Proliferation Kit with Alexa Fluor 647, Mitochondrial Permeability Transition Pore Assay Kit or MPTP Assay Kit, Mito‐Tracker Deep Red FM, Intracellular Iron Colorimetric Assay Kit, and Lipid Peroxidation MDA Assay Kit were purchased from Beyotime, China. Anti‐gamma H2A.X antibody was purchased from Abcom. GPX4 Monoclonal antibody was purchased from Proteintech. 8‐Hydroxydeoxyguanosine ELISA Kit was purchased from Elabscience, China.

*Synthesis of NH_2_‐MIL‐101(Fe)*. In 30 ml of DMF was dissolved 453 mg of terephthalic acid (2.5 mmol) and 1.35 g of ferric chloride hexahydrate (5 mmol). The mixture solution was poured into a 100 ml Teflon‐lined autoclave and heated at 110 °C for 24 h. The solid product obtained was collected by centrifugation, washed with DMF and ethanol, and dried overnight in a vacuum oven at 80 °C.

*Synthesis of GEM‐DVDMS@NH_2_‐MIL‐101(Fe)*. Deionized water (10 ml) was mixed with 25 mg of NH_2_‐MIL‐101(Fe) under stirring to form an even suspension. Next, 5 mg of GEM and 5 mg of DVDMS were added and stirred at room temperature for 12 h. The reactions were performed in the dark. Subsequently, the product was collected by centrifugation, washed twice with deionized water to remove redundant GEM and DVDMS, and dried overnight under a vacuum.

*Synthesis of GEM‐DVDMS@NH_2_‐MIL‐101(Fe)@TpPa‐1*. It all started with dissolving 30 mg of GEM‐DVDMS@NH2‐MIL‐101(Fe) in a mixture of 1.5 ml mesitylene and 1.5 ml 1,4‐dioxane. A 10 ml Schlenk tube was used for the reaction. After 30 min, the mixture was sonicated to obtain a uniform suspension. The above solution was then supplemented with 15.75 mg of Tp and 12 mg of Pa‐1. Three cycles of freeze‐thaw degassing in liquid nitrogen were followed by heating the mixture to 120 °C for 72 h, cooling it to room temperature, filtering it, and washing it with tetrahydrofuran (THF). The GEM‐DVDMS@NH2‐MIL‐101(Fe)@TpPa‐1 was stabilized by 12 h of hoover drying at 120 °C. UV–vis absorption spectra at wavelengths of 630 nm, and 269 nm were used to determine DVDMS and GEM of the drug‐loading efficiency (DE) and encapsulation efficiency (EE) of the nanoparticles.^[^ ^48^ ^]^

The *DE* and *EE* were computed using the following formula:

DE =$\frac{weight\;of\;the\;GEM\;or\;DVDMS\;in\;the\;GDMCN2}{weight\;of\;the\;GDMCN2}\;$×100%

EE =$\frac{weight\;of\;the\;GEM\;or\;DVDMS\;in\;the\;GDMCN2}{threoretic\;weight\;of\;the\;GEM\;or\;DVDMS\;in\;the\;GDMCN2\;}\;$ ×100%

### Analysis and Characterization

SEM and TEM (Leica, Germany) were used to examine the morphology, size, and microstructure of the samples at an electron emission gun operating voltage of 300 kV. Fourier transform infrared spectra were acquired using an infrared spectrometer (Nicolet iN10 MX; Thermo Fisher Scientific, Inc.). The polydispersity index and mean hydrodynamic particle size were determined by dynamic light scattering (DLS; Brookhaven Instruments Omni). The near‐infrared radiation (NIR) absorption peaks were obtained by measuring the UV–vis spectra of the materials using a Cary 5000 UV—vis–NIR spectrophotometer. Brunauer‐Emmett‐Teller (BET; Mac ASAP2460) was used.

### Analysis of Targeting Ability and Cellular Uptake

To test the cell capacity for targeting, PANC‐1/GEM cells were seeded in a confocal dish and allowed to grow until they reached 80% confluency. After incubating the dish for 30 min, TRITC‐labeled NRP2 mAb, GDNC, and GDMCN2 (12.5 µg mL^−1^) was added. Cell nuclei were stained with Hoechst 33342. CLSM observations allowed to ascertain whether GDMCN2 targets PANC‐1/GEM cells. When the PANC‐1/GEM cells reached 80% confluence in a confocal dish, they were treated with TRITC‐labeled GDMCN2 to induce phagocytosis. The cells were analyzed at the beginning, middle, and end of the incubation period. The phagocytic rate of GDMCN2 in PANC‐1/GEM cells was determined after nuclear labeling with Hoechst 33342 and subsequent observation using CLSM. After the PANC‐1/GEM cells reached 80% confluence in a confocal plate, they were seeded with TRITC‐labeled GDMCN2 and allowed to co‐localize for 12 h. Green fluorescent probes were used to localize and image the ER, mitochondria, and lysosomal organelles in PANC‐1/GEM cells to analyze their colocalization. These probes included ER‐Tracker Green (BODIPY FL Glibenclamide), MitoTracker Green (Benzoxazolium), and LysoSensor Green DND‐189. CLSM was used to quantify the rate of cellular absorption and TEM was used to study the intracellular localization and distribution of GDMCN2 in PANC‐1/GEM cells.

### Cell Transmission Electron Microscopy

PANC‐1/GEM cells (6 × 10^5^ per well in a six‐well plate) were treated with 20 µg mL^−1^ with GDMCN2 in 2.5 mL for 12 h. After rinsing the samples with PBS to remove free nanoparticles, Overnight fixation of cells with 2.5% glutaraldehyde at 4 °C, and then dehydrated in ethanol (50, 70, 90, and 100% solutions). An ultrathin microtome was used to slice the cellular samples, which were then examined by TEM.

### In Vitro Cytotoxicity Evaluation

High‐glucose Dulbecco's Modified Eagle Medium (DMEM) with 1% penicillin‐streptomycin and 10% fetal bovine serum (FBS) was used to cultivate HPDE6C7 and PANC‐1/GEM cells obtained from the American Type Culture Collection. The CCK‐8 assay (Yeason Biotechnology, China) was used to determine cell viability according to the manufacturer's protocol. Overnight, 96‐well plates were seeded with 5 × 10^3^ HPDE6C7 or PANC‐1/GEM cells. After the cells were treated with the medicines, those in the US group were blasted for 2 min with US at 1 W cm^−2^. Cell viability was determined by measuring the optical density (OD) of each well at 450 nm using a SpectraMax microplate reader (Model 680; Bio‐Rad Laboratories, Inc., Tokyo, Japan).

Following seeding, the PANC‐1/GEM cells were treated with GDMCN2 for 12 h at 80% confluence in 6‐well plates. After 15 min in the dark, the cells were stained with Annexin V‐FITC/PI and analyzed by flow cytometry. Cell viability was determined using calcein‐AM/PI staining. PANC‐1/GEM cells were plated in a 6‐well plate and treated with GDMCN2 for 12 h once confluence reached 80%. Subsequently, the cells were exposed to different treatments, one of which was ultrasonic therapy (1 W cm^−2^, 2 min). The cells were incubated for an additional 4 h, stained with calcein‐AM/PI for 25 min at room temperature in the dark, washed with PBS, and analyzed using CLSM. The concentration of GDMCN2 nanocages is 20 µg mL^−1^ (equal to 2.88 µg mL^−1^ of DVDMS and 4 µg mL^−1^ of GEM).

### 3D Cell Culture

To ensure minimal adsorption, ultralow‐adsorption culture plates were used for cell culture. Once the cells formed round spheres, they were treated with different drugs based on the assigned groups. After a 12 h incubation, images of the cell spheres in each group were captured, and the US group was subjected to ultrasonic treatment at the same power as before. Following treatment, the cells were incubated for an additional 6 h, after which images were captured, and the CellTiter‐Lum Luminescence 3D Cell Viability Detection Kit was used to evaluate cell viability in different treatment groups.

### In Vitro Oxidative Stress

First, PANC‐1/GEM cells were seeded in a confocal dish until the cell density reached 90% confluence. Next, the different drugs were added according to the experimental design and incubated for 12 h. Next, the cells were treated with US and incubated for an additional 6 h. To assess intracellular ROS levels, mitochondrial membrane potential, MPTP, mitochondrial calcium ions, and mitochondrial mass, DCFH‐DA, JC‐1, Rhod‐2, AM, and MitoTracker Deep Red FM fluorescent probes were used according to the manufacturer's instructions. Nuclei were stained with Hoechst 33342. Finally, CLSM or flow cytometry was used to detect and analyze the results.

In Vitro Proliferation Analysis

First, PANC‐1/GEM cells were seeded in a confocal dish and incubated until the cell density reached 90%. Different drugs were added according to the specific treatment group and incubated for 12 h before US treatment. After 6 h, the BeyoClic EdU Cell Proliferation Kit with Alexa Fluor 647, CFDA SE Cell Proliferation and Cell Tracking Kit, or Cell Cycle and Apoptosis Analysis Kit were used according to the manufacturer's instructions to detect the cell proliferation ability of each treatment group. Finally, CLSM or flow cytometry was used for analysis.

### Single‐Cell Gel Electrophoresis

PANC‐1/GEM cells were treated with drugs and US, and single‐cell gel electrophoresis was performed to evaluate DNA damage. The cells were mixed with agarose and lysed and then placed in an electrophoresis tank for 20–30 min. CLSM observations and image analysis were used to evaluate DNA damage.

8‐OHdG ELISA Analysis

PANC‐1/GEM cells were seeded in confocal dishes and treated with different drugs for 12 h, followed by US treatment. After 6 h, the cell supernatant was collected and analyzed using an 8‐OHdG ELISA Kit following the manufacturer's instructions.

### Western Blotting

Electrophoretically separated cell lysates were deposited onto polyvinylidene difluoride (Bio‐Rad) membranes. Following overnight incubation at 4 °C with primary antibodies, membranes were blocked with Tris‐buffered saline (TBS) containing 5% skim milk. After three washes with Tris‐buffered saline with 0.1% Tween 20 detergent (TBST) buffer, the membranes were incubated with secondary antibodies at room temperature for 45 min. The ChemDoc Imaging System (Bio‐Rad) was used to capture images after five washes in TBST.

### Immunofluorescence Staining

PANC‐1/GEM cells were grown in 12‐well plates to achieve ≈80% confluence. Then, the participants were divided into groups and administered either a placebo or medication; the US group received US therapy for 2 min using a 1 W cm^−2^ machine. The cells were permeabilized with 0.2% Triton X‐100 on ice for 20 min after being fixed with 4% paraformaldehyde overnight. This was followed by three 10‐min washes in 0.01 m phosphate‐buffered saline. Rabbit anti‐GPX4 (1:200; Abcam) and rabbit anti–H2A.X (1:100; Abcam) were incubated with the cells overnight at 4 °C after being blocked with 2% bovine serum albumin at room temperature for 1 h and rinsed with PBS three times for 10 min each. The cells were treated overnight at room temperature with Alexa Fluor 488 donkey anti‐rabbit antibody (1:1000; Abcam) and washed three times with 0.01 m PBS the following day. The cells were fixed in a medium containing 4′,6‐diamino‐2‐phenylindole (DAPI), and CLSM was used to reconstruct them in three dimensions after three washes in PBS.

### Quantitative Real‐Time PCR

Trizol was used to extract total RNA from PANC‐1/GEM cells, and the First Strand cDNA Synthesis SuperMix for qPCR (Invitrogen, Carlsbad, CA) was used to synthesize complementary DNA in preparation for quantitative polymerase chain reaction. Following this, RT‐qPCR was carried out in a total volume of 25 µL in accordance with the manufacturer's instructions. The following primer sets were used for RT‐qPCR:

**Table:** 

Human β‐actin forward:	5′‐ GGCACCCAGCACAATGAAG −3′;
Human β‐actin reverse:	5′‐ CCGATCCACACGGAGTACTTG −3′;
Human ERN1 forward:	5′‐ GCGAACAGAATACACCATCAC −3′;
human ERN1 reverse:	5′‐ ACCAGCCCATCACCATTG −3′;
Human EIF2AK3 forward:	5′‐ GAACCAGACGATGAGACAGAG −3′;
Human EIF2AK3 reverse:	5′‐ GGATGACACCAAGGAACCG −3′;
Human ATF6 forward:	5′‐ CCTGTCCTACAAAGTACCATGAG −3′;
Human ATF6 reverse:	5′‐ CCTTTAATCTCGCCTCTAACCC −3′;
Human HSPA5 forward:	5′‐ CTGCCATGGTTCTCACTAAAATG −3′;
Human HSPA5 reverse:	5′‐ TTAGGCCAGCAATAGTTCCAG −3′;
Human XBP1 forward:	5′‐ GCCCTGGTTGCTGAAGAG −3′;
Human XBP1 reverse:	5′‐ AGTCAATACCGCCAGAATCC −3′;
Human DDIT3 forward:	5′‐ TGACCAGGGAAGTAGAGGC −3′;
Human DDIT3 reverse:	5′‐ AGTGAGAGGGTAGTCAGTAGC −3′;
Human SHC1 forward:	5′‐ CCAGCAGGCAGAGAGCTTTT −3′;
Human SHC1 reverse:	5′‐ TCCATGCTACTCCCAGCTCT −3′;
Human PRDX3 forward:	5′‐ GTCTTGCACTAAGATCAAGCCA −3′;
Human PRDX3 reverse:	5′‐ AAACTAGCTAGCCAGCCACC −3′;
Human MICU1 forward:	5′‐ TGGAAAGAAAATTTCCCAGGAACG −3′;
Human MICU1 reverse:	5′‐ GACGATCTCTGTGGCGCATA −3′;
Human PCBP2 forward:	5′‐ CCTTTTCCCCTCAGTCGC −3′;
Human PCBP2 reverse:	5′‐ ATCCACCTTCAATCACACCG −3′;
Human ATG7 forward:	5′‐ TTTTGCTATCCTGCCCTCTG −3′;
Human ATG7 reverse:	5′‐ GCTGTGACTCCTTCTGTTTGAC −3′;
Human NCOA4 forward:	5′‐ TTGAGGTGTAGTGATGCACG −3′;
Human NCOA4 reverse:	5′‐ CTAAGACATTCCAGGTGACGG −3′;
Human GPX4 forward:	5′‐ CCTTCCCGTGTAACCAGTTC‐3′;
Human GPX4 reverse:	5′‐ TCTTCATCCACTTCCACAGC‐3′;
Human hENT1 forward:	5′‐ CCACTCTATCAAAGCCATCCTG −3′;
Human hENT1 reverse:	5′‐ ATGAAGTAACGTTCCCAGGTG‐3′;
Human hCNT3 forward:	5′‐ TTCGGTGGGCTCATAATGTAC −3′;
Human hCNT3 reverse:	5′‐ GCTATAAATCCAGGGTCAGTCC −3′;
Human MUC4 forward:	5′‐ GTCCTATGCCCTGTTTCTCTAC‐3′;
Human MUC4 reverse:	5′‐ CGATACCTCTCCCACACTG −3′;
Human MRP5 forward:	5′‐ CAGAGACCGTGAAGATTCCAAG −3′;
Human MRP5 reverse:	5′‐ TGAGCTGAGAATGCATGGAG −3′;
Human dCK forward:	5′‐ TTTATCTTCAAGCCACTCCAGAG −3′;
Human dCK reverse:	5′‐ GTTGGTTTTCAGTGTCCTATGC −3′;
Human RRM1 forward:	5′‐ ACCGCCCACAACTTTCTAG −3′;
Human RRM1 reverse:	5′‐ CCAGTAGCCCGAATACAACTC −3′;
Human CDA forward:	5′‐ AAGGGTACAAGGATTTCAGGG −3′;
Human CDA reverse:	5′‐ ACAATATACGTACCATCCGGC‐3′;
Human ERCC1 forward:	5′‐ AATTTGTGATACCCCTCGACG −3′;
Human ERCC1 reverse:	5′‐ TGTGAGATGGCATATTCGGC −3′;
Human TOP1 forward:	5′‐ CTGTAGCCCTGTACTTCATCG −3′;
Human TOP1 reverse:	5′‐ CTGTAGCCCTGTACTTCATCG‐3′;
Human MCL‐1 forward:	5′‐ TCTTCCCCAGTTTTCTCAGC −3′;
Human MCL‐1 reverse:	5′‐ ACAGTAGAGGTTGAGTCCGA −3′;
Human BAX forward:	5′‐ GACATGTTTTCTGACGGCAAC −3′;
Human BAX reverse:	5′‐ AAGTCCAATGTCCAGCCC −3′;
Human BAK forward:	5′‐ AGAGTTCCAGACCATGTTGC −3′;

**Table:** 

Human BAK reverse:	5′‐ GTAGCCGAAGCCCAGAAG −3′;
Human LAT2 forward:	5′‐ GAAGCCTACATAGACCCCATTG −3′;
Human LAT2 reverse:	5′‐ CTCTGTGGTTTTCTGCTTGC −3′;
Human FASN forward:	5′‐ CAAGCTGAAGGACCTGTCTAG −3′;
Human FASN reverse:	5′‐ CGGAGTGAATCTGGGTTGATG −3′;
Human MYC forward:	5′‐ TTCGGGTAGTGGAAAACCAG −3′;
Human MYC reverse:	5′‐ AGTAGAAATACGGCTGCACC −3′;
Human EGFR forward:	5′‐ AAGCCATATGACGGAATCCC −3′;
Human EGFR reverse:	5′‐ GGAACTTTGGGCGACTATCTG −3′;
Human TP53 forward:	5′‐ GCCATCTACAAGCAGTCACAG −3′;
Human TP53 reverse:	5′‐ GCCATCTACAAGCAGTCACAG −3′;
Human TGF‐β forward:	5′‐ GCCTTTCCTGCTTCTCATGG −3′;
Human TGF‐β reverse:	5′‐ TCCTTGCGGAAGTCAATGTAC −3′;

Human actin was amplified as an internal reference using the same procedure for each sample. Each experiment was repeated thrice.

### Detection of Intracellular Lipid Oxidation and Antioxidant Capacity

PANC‐1/GEM cells (6 × 10^5^ each well, 6‐well dish) were treated with GDMCN2 (20 µg mL^−1^) for 12 h, followed by exposure to a US treatment machine (1 W cm^−2^, 2 min). After further incubation for 6 h, changes in intracellular antioxidant capacity were detected using the GSH and GSSG Assay Kit and Total Antioxidant Capacity Assay Kit with the ABTS method, following the manufacturer's instructions. An Intracellular Iron Colorimetric Assay Kit was used to detect changes in intracellular Fe^3+^ content, while the degree of lipid peroxidation was determined using BODIPY C11 and Liperfluo fluorescent probes. The mean fluorescence intensity after treatment was analyzed by CLSM. The Lipid Peroxidation MDA Assay Kit was used for the quantitative detection of the degree of lipid peroxidation.

### Hemolysis Assay

Mouse blood was collected via orbital bleed into a microcentrifuge tube containing 20 µL of 10% EDTA and 10 mL PBS. The tube was centrifuged at 1500 rpm for 15 min to separate the plasma and buffy coat layers. Red blood cells (RBCs) were isolated and washed several times with PBS until the upper solution was devoid of red coloration. The obtained RBCs were suspended in 200 µL PBS and further diluted with 9.8 mL PBS. Subsequently, 0.5 mL of the RBCs suspension was combined with 0.5 mL PBS containing varying concentrations of GDMCN2 nanoparticles (100, 400, and 800 µg mL^−1^). As a control, 0.5 mL of the RBCs suspension was incubated with PBS (0.5 mL) and water (0.5 mL) separately. The solutions were gently mixed and incubated at 37 °C for 3 h. Following incubation, RBCs were collected by centrifugation at 10 000 rpm for 3 min and the absorbance of the supernatant at 576 nm was measured using a microplate reader. The percentage of hemolysis was calculated using the following formula:

(1) $$\begin{array}{ccc} \mathrm{Hemolysis}\left(\%\right) & = & \left[\left(\mathrm{Sample}\;\mathrm{absorbance}-\mathrm{Background}\;\mathrm{absorbance}\right)/\right.\\ & & \left.\left(\mathrm{Positive}\;\mathrm{control}-\mathrm{Negative}\;\mathrm{control}\right)\right]\times 100\% \end{array}$$

### In Vivo Targeting and Imaging

To create a subcutaneous tumor model, female BALB/c nude mice (6‐8 weeks old) were obtained from the Xiamen University Experimental Animal Center. Mice were subcutaneously injected with PANC‐1/GEM cells (1×10^6^ cells in 100 µL). Once the tumor volumes reached 100 mm^3^, the mice were injected intravenously with 20 mg kg^‐1^ TRITC‐labeled GDMC or GDMCN2 and 3mg kg^‐1^ DVDMS. Following anesthesia, the mice were examined at 1, 4, 8, 12, 24, 48, and 72 h using PerkinElmer IVIS Lumina III small animal in vivo optical imaging system. Tumors and major organs were harvested 72 h after the mice were injected with fluorescent tags, and the animals were euthanized for ex vivo fluorescence imaging.

### In Vivo Antitumor Action of SDT

When the average tumor volume reached 100 mm^3^, a subcutaneous tumor model was created in female BALB/c nude mice to test the anticancer effects of SDT on GDMCN2. Tumor‐bearing mice were randomized into seven treatment groups: i) PBS, ii) US, iii) GEM, iv) DVDMS, v) DVDMS+US, vi) GDMCN2, vii) GDMCN2+US. The mice received tail vein injections of 100 L of each drug: 20 mg kg^−1^ GDMCN2, 3 mg kg^−1^ DVDMS, and 4 mg kg^−1^ GEM. The tumor locations of the mice were treated with US at a frequency of 1.0 MHz, an intensity of 1.5 W cm^−2^, 6 min. The mice were weighed twice daily, and the *L* and *W* dimensions of the tumors were assessed using a vernier caliper and a microelectronic scale. *V* = *L × W* ^2^/2 was used to calculate the tumor volume. Tumor tissues and major organs were harvested from the mice that had perished after 14 days of treatment. Tumor tissues were used for γ‐H2A.X and GPX4 immunofluorescent staining experiments, HE staining, and ki67 immunohistochemistry staining. Strict measures were implemented in the in vitro sonodynamic evaluation experiment, including conducting the operation in a controlled dark room to eliminate the impact of light and selecting an ultrasonic power with a low heat output. These measures were taken to ensure the reliability and scientific rigor of this study.

### In Vivo Biosafety Assessment

The obtained organs were fixed, and HE was applied to the tissue slices. Blood was drawn from each group, and a standard blood analysis was performed.

All animal experiments were approved by the Xiamen University Ethics Committees (XMULAC20170297).

### Statistical Analysis

All values are expressed as mean ± SD, and the significance of the data is based on Student's *t*‐test, one‐way analysis of variance (ANOVA), and two‐way ANOVA (*p < 0.05, **p < 0.01, ***p < 0.001 and ****p < 0.0001).

## Conflict of Interest

The authors declare no conflict of interest.

## Supporting information

Supporting Information

## Data Availability

The data that support the findings of this study are available in the supplementary material of this article.
