# Supplementary material for: Sonodynamic Therapy of NRP2 Monoclonal Antibody‐Guided MOFs@COF Targeted Disruption of Mitochondrial and Endoplasmic Reticulum Homeostasis to Induce Autophagy‐Dependent Ferroptosis
Source: Adv Sci (Weinh). 2023 Sep 3;10(30):2303872. doi: 10.1002/advs.202303872 (PMC10602529; doi:10.1002/advs.202303872)
Supplement: Supplementary file 1 — Supporting Information [file ADVS-10-2303872-s001.pdf]

ADVANCED SCIENCE
Open Access

Supporting Information

for *Adv. Sci.*, DOI 10.1002/advs.202303872

Sonodynamic Therapy of NRP2 Monoclonal Antibody-Guided MOFs@COF Targeted Disruption of Mitochondrial and Endoplasmic Reticulum Homeostasis to Induce Autophagy-Dependent Ferroptosis

Zhiyu Zhao, Yanjie Wu, Xiaochen Liang, Jiajing Liu, Yi Luo, Yijia Zhang, Tingting Li, Cong Liu, Xian Luo, Jialin Chen, Yunjie Wang, Shengyu Wang, Ting Wu, Shaoliang Zhang, Dong Yang, Wengang Li, Jianghua Yan, Zhihai Ke and Fanghong Luo**

Supporting Information

Sonodynamic Therapy of NRP2 Monoclonal Antibody-Guided MOFs@COF Targeted Disruption of Mitochondrial and Endoplasmic Reticulum Homeostasis to Induce Autophagy-Dependent Ferroptosis

Zhiyu Zhao[a#], Yanjie Wu[b#], Xiaochen Liang[c], Jiajing Liu[a], Yi Luo[d], Yijia Zhang[a], Tingting Li[a], Cong Liu[a], Xian Luo[a], Jialin Chen[d], Yunjie Wang[a], Shengyu Wang[a], Ting Wu[a], Shaoliang Zhang[e], Dong Yang[a], Wengang Li[a], Jianghua Yan[a], Zhihai Ke[b], Fanghong Luo[a]**

Zhiyu Zhao, Jiajing Liu, Yijia Zhang, Tingting Li, Cong Liu, Xian Luo, Yunjie Wang, Shengyu Wang, Ting Wu, Dong Yang, Wengang Li, Jianghua Yan, Fanghong Luo
Cancer Research Center, School of Medicine, Xiamen University, Xiamen, 361000, P. R. China
Fanghong Luo(luofanghong@xmu.edu.cn)

Yanjie Wu, Zhihai Ke
School of Science and Engineering, Shenzhen Key Laboratory of Innovative Drug Synthesis, The Chinese University of Hong Kong, Shenzhen, 518172, P. R. China
Zhihai Ke(kezhihai@cuhk.edu.cn)

Xiaochen Liang
Environmental Toxicology, University of California, Riverside, California, 92507, United States

Yi Luo, Jialin Chen
School of Basic Medicine,School of Clinical Medicine,Fujian Medical University, Fuzhou, 350122, P. R. China

Shaoliang Zhang
Shanghai Guangsheng Biopharmaceutical Co.,Ltd, Shanghai， 200120, P. R. China

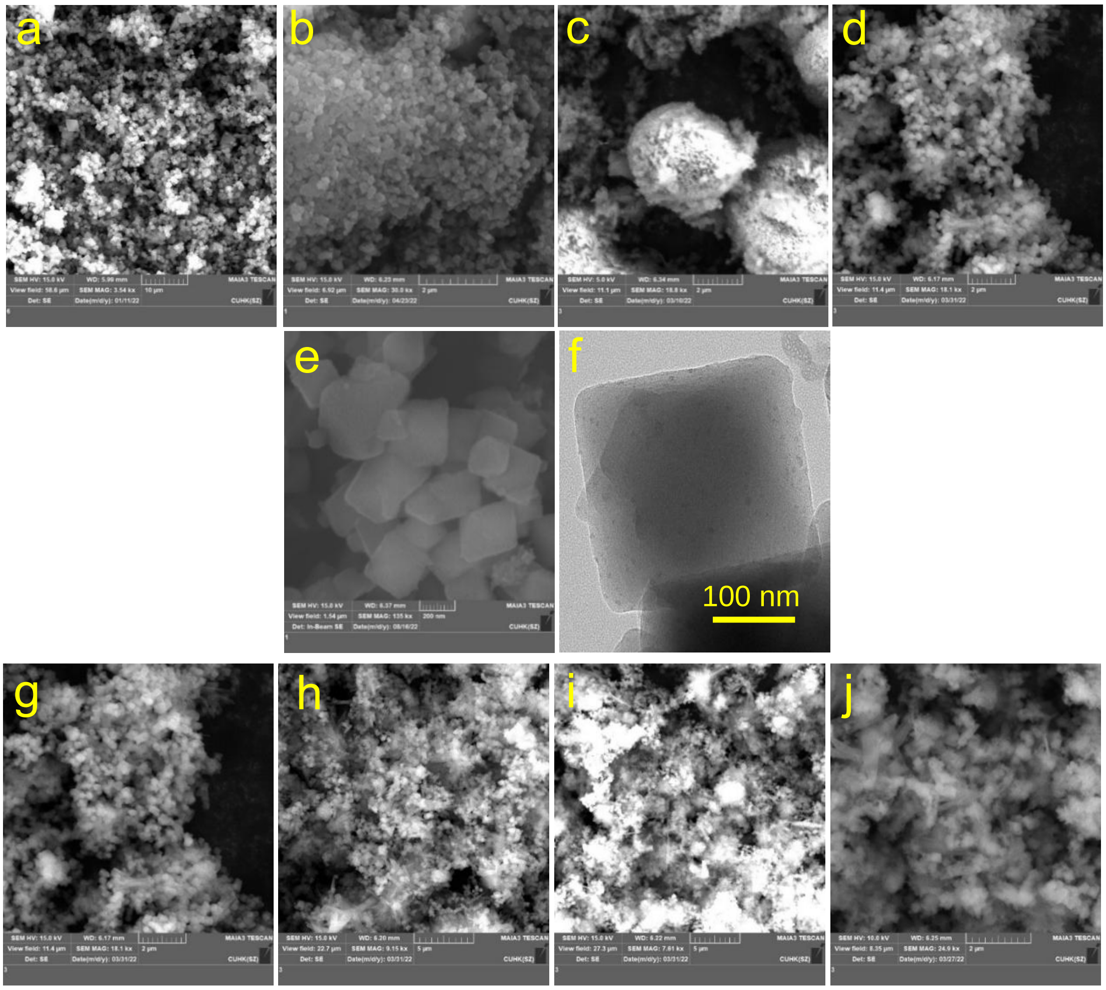

Fig. S1 SEM images of a) NH_2-MIL-101 (Fe), b) GEM-DVDMS@NH_2-MIL-101 (Fe), c) TpPa-1, (d) NH_2-MIL-101 (Fe)@TpPa-1, e) NRP2-GEM-DVDMS/NH_2-MIL-101 (Fe)@TpPa-1, f) TEM image of NH_2-MIL-101(Fe). Different weight ratios of NH2-MIL-101 (Fe)@TpPa-1: (g) 15.75 mg Tp, 12 mg Pa-1, (h) 31.5 mg Tp, 24 mg Pa-1, i) 47.25 mg Tp, 36 mg Pa-1, j) 63mg Tp, 48 mg Pa-1, samples were denoted as NH2-MIL-101(Fe)@TpPa-1-1, NH2-MIL-101(Fe)@TpPa-1-2, NH2-MIL-101(Fe)@TpPa-1-3, NH2-MIL-101(Fe)@TpPa-1-4, respectively. The weight of NH2-MIL-101(Fe) was fixed at 30 mg.

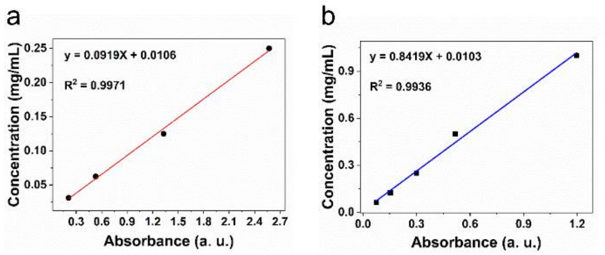

Fig. S2 a) Standard curve of GEM at different concentrations. b) Standard curve of DVDMS at different concentrations.

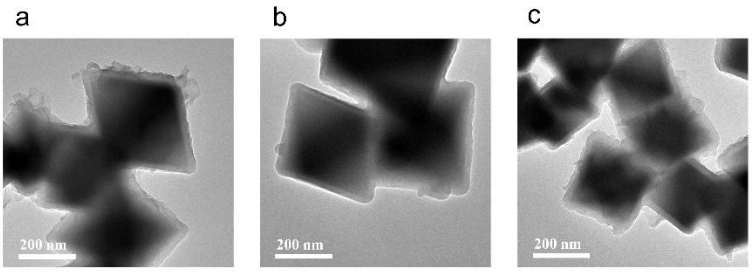

Fig. S3 TEM image of GDMCN2 stability in PBS. a) 24 h. b) 72h. c) 168 h.

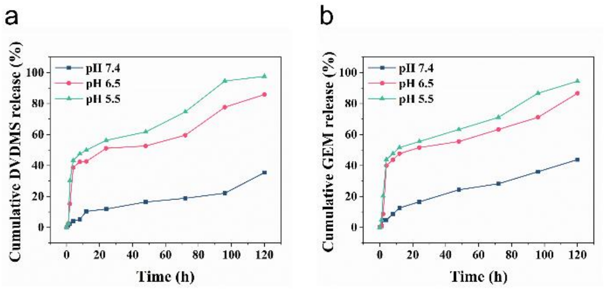

Fig. S4 *In vitro* cumulative release profiles of a) DVDMS and b) GEM.

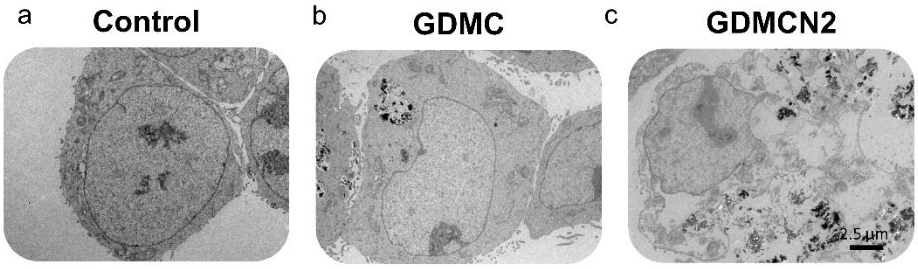

Fig. S5 Intracellular TEM images of PANC-1/GEM cells incubated with a) PBS b) GDMC, and c) GDMCN2 following 1 h of incubation.

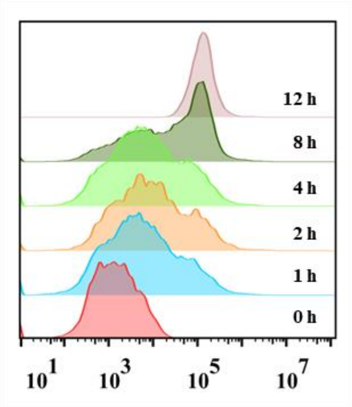

Fig. S6 Flow cytometric analysis of PANC-1/GEM cells following incubation with GDMCN2 for 0h, 1h, 2h, 4h, 8h, and 12h.

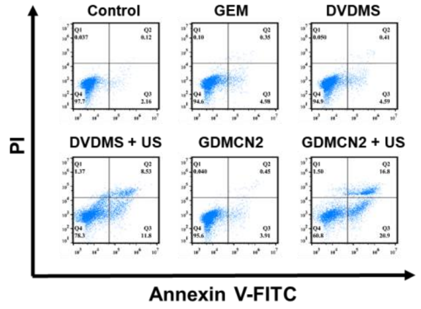

Fig. S7 Flow cytometer analysis after various treatments.

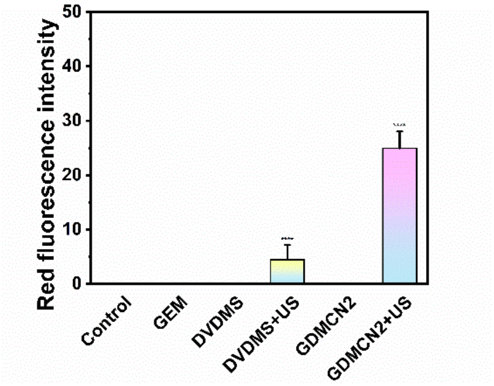

Fig. S8 Red fluorescence intendity in PANC-1/GEM cells stained with AM-PI under different treatment.

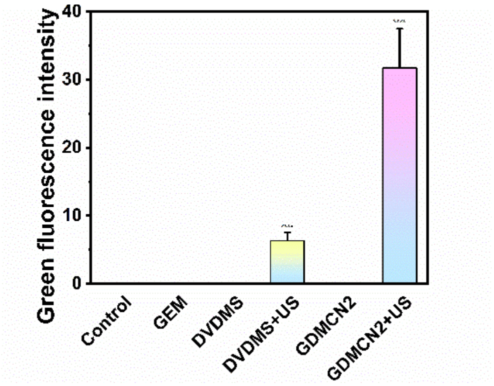

Fig. S9 Green fluorescence intendity in PANC-1/GEM cells stained with DCFH-DA under different treatment.

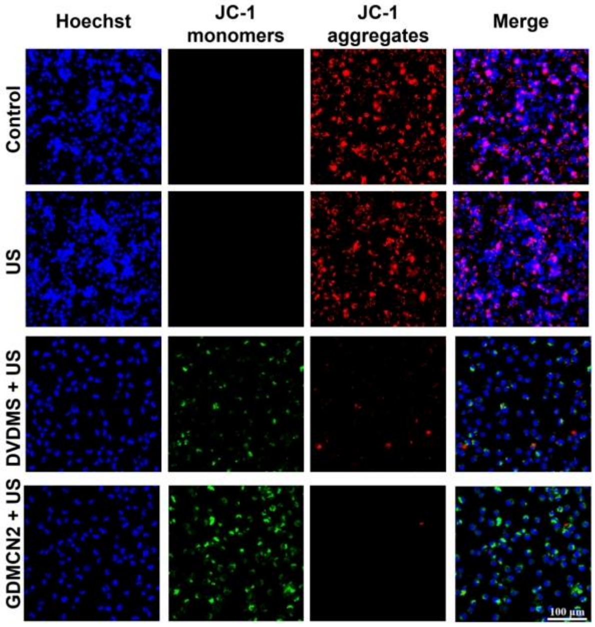

Fig. S10 CLSM images of PANC-1/GEM cells stained with JC-1 following Control, US, DVDMS+US, and GDMCN2+US group.

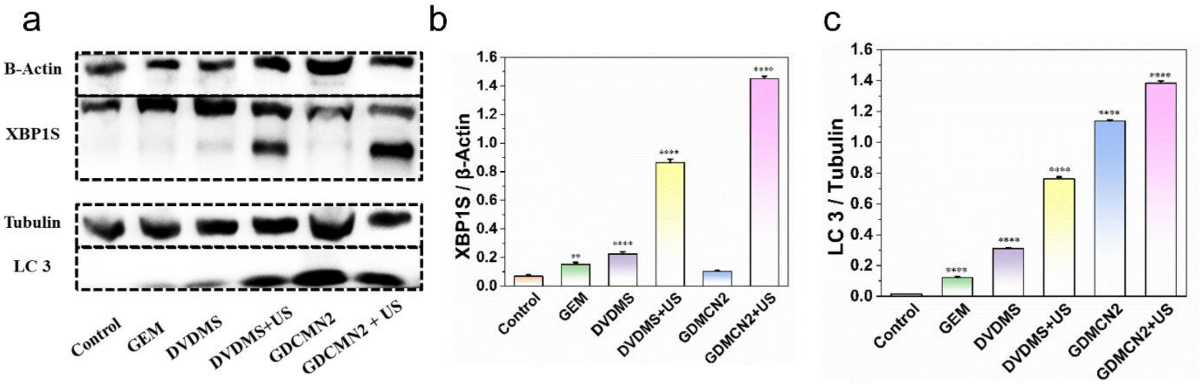

Fig. S11 a) Protein expression levels of XPB1S and LC3 in PANC-1/GEM cells with different treatments. b and c) Quantitative analysis of XBP1S and LC3 proteins

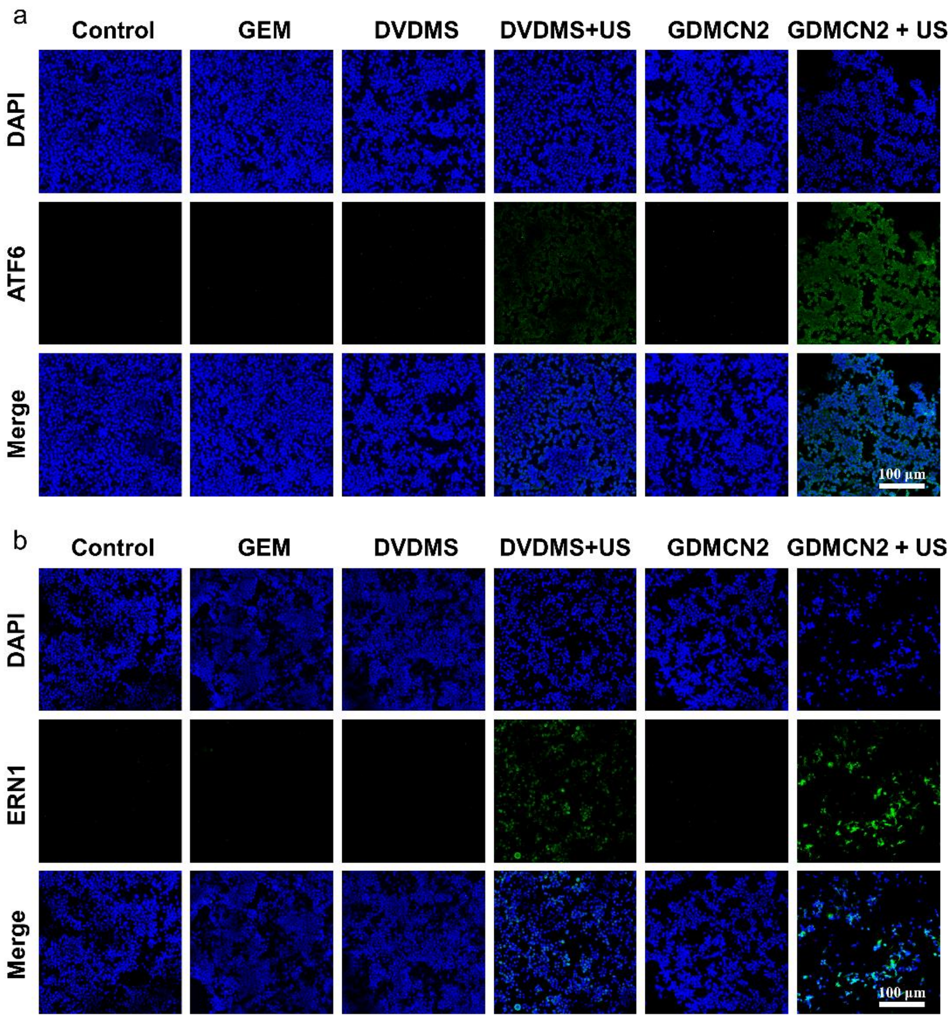

Fig. S12 CLSM image of cell immunofluorescence in PANC-1/GEM cells treated with various treatments. a) ATF6 protein, b) ERN1 protein.

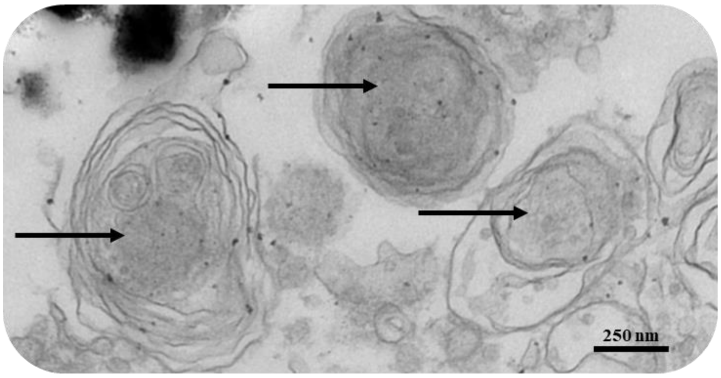

Fig. S13 Autophagy-lysosomes in TEM images of PANC-1/GEM cells after GDMCN2 combined with US treatment.

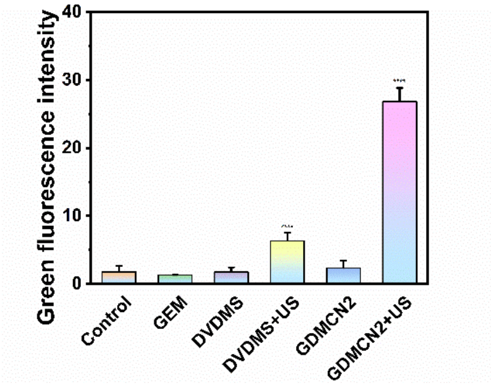

Fig. S14 Green fluorescence intendity in PANC-1/GEM cells stained with γ-H2A.X antibody under different treatment.

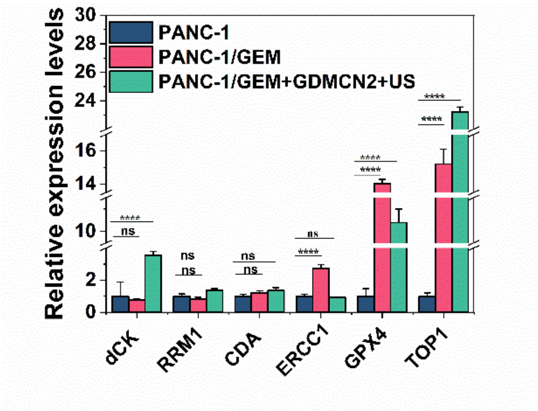

Fig. S15 RT-qPCR relative expression levels of DNA repair-related genes.

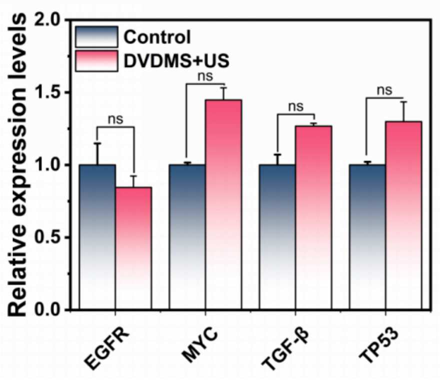

Fig. S16 RT-qPCR relative expression levels of cell proliferation-related genes.

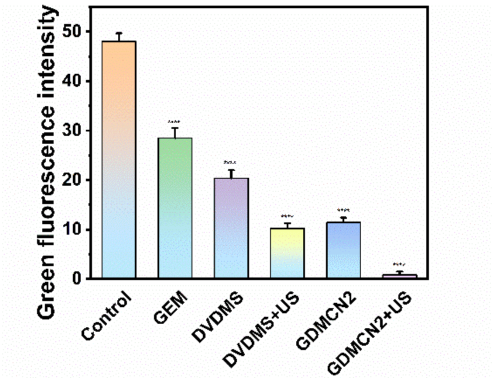

Fig. S17 Green fluorescence intendity in PANC-1/GEM cells stained with ThiolTracker Violet under different treatment.

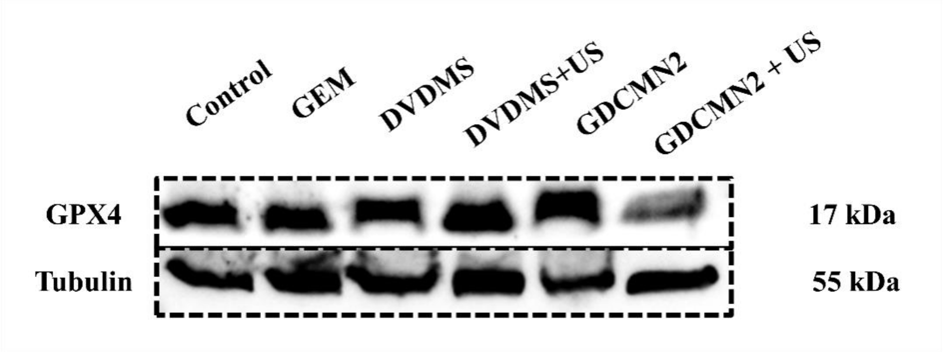

Fig. S18 Ferroptosis-associated Western Blot results.

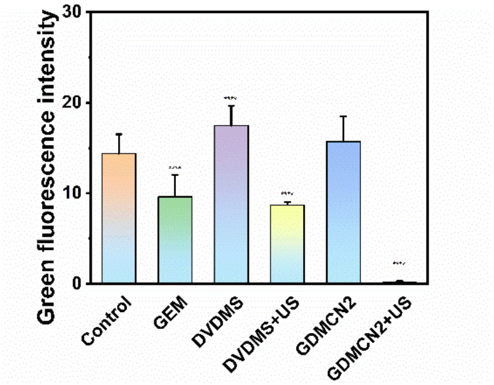

Fig. S19 Green fluorescence intendity in PANC-1/GEM cells stained with GPX4 antibody under different treatment.

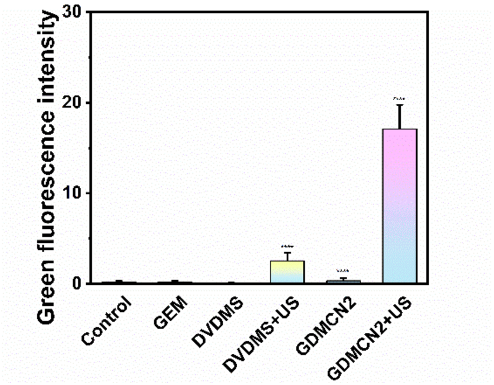

Fig. S20 Green fluorescence intendity in PANC-1/GEM cells stained with BODIPY C11 under different treatment.

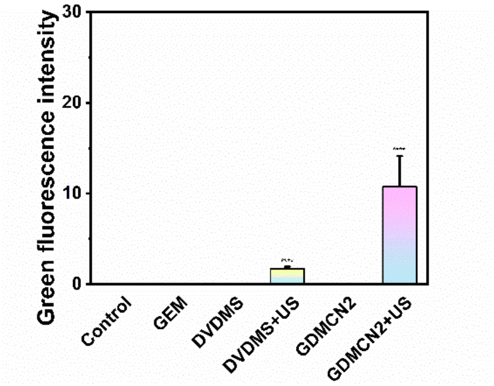

Fig. S21 Green fluorescence intendity in PANC-1/GEM cells stained with LiperFluo under different treatment.

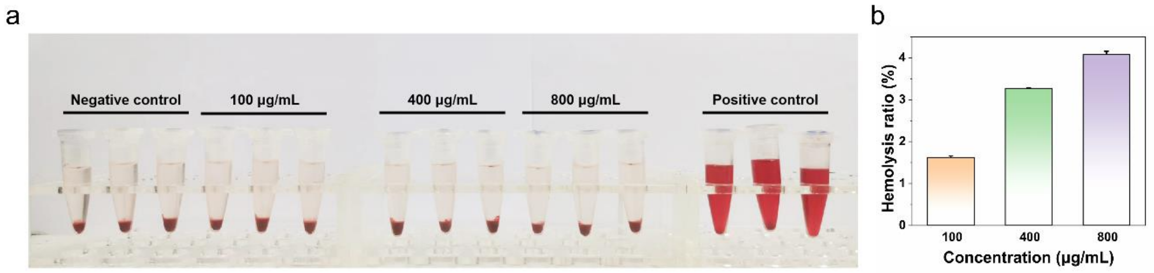

Fig. S22 a and b)Hemolysis assay of GDMCN2 in different concentration.

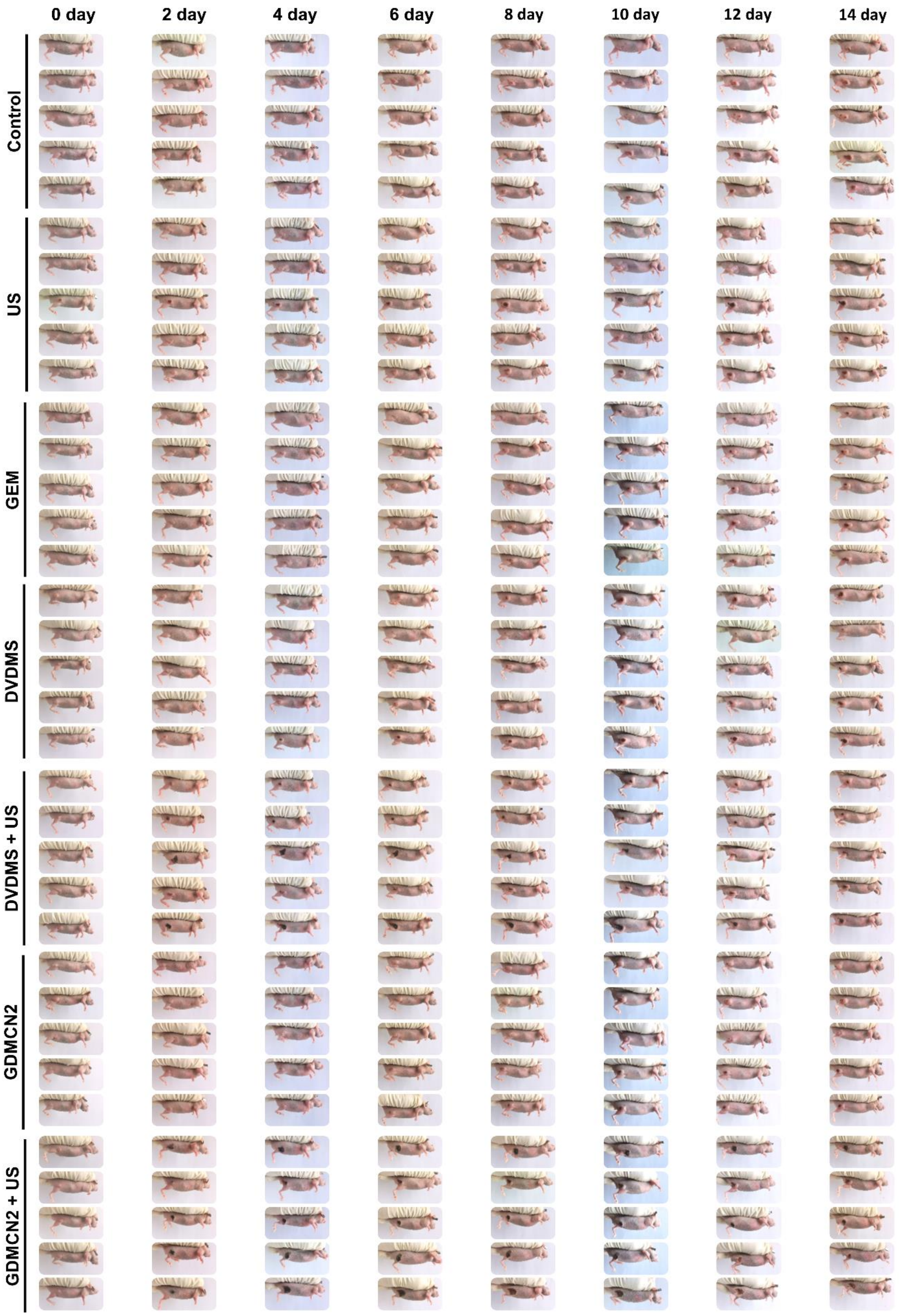

Fig. S23 Representative digital images of mice from each group within 14 days.

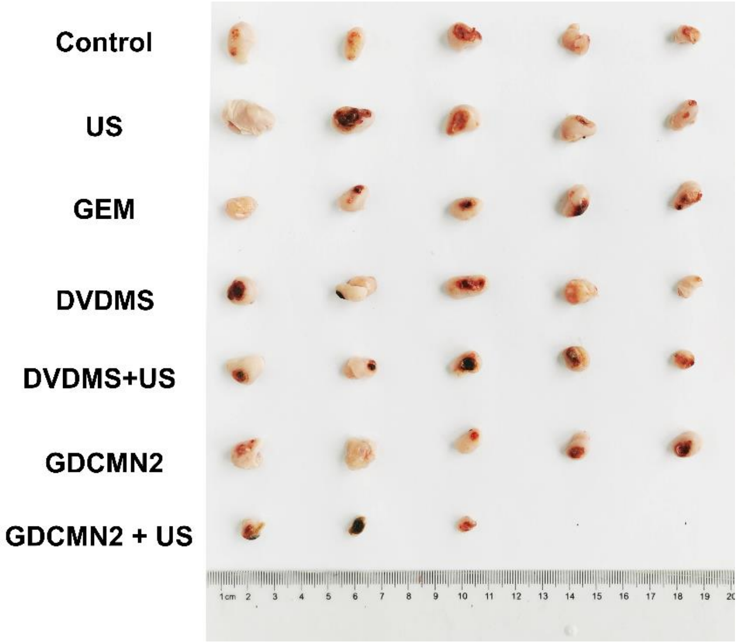

Fig. S24 Tumor tissue isolated from different treatment groups.

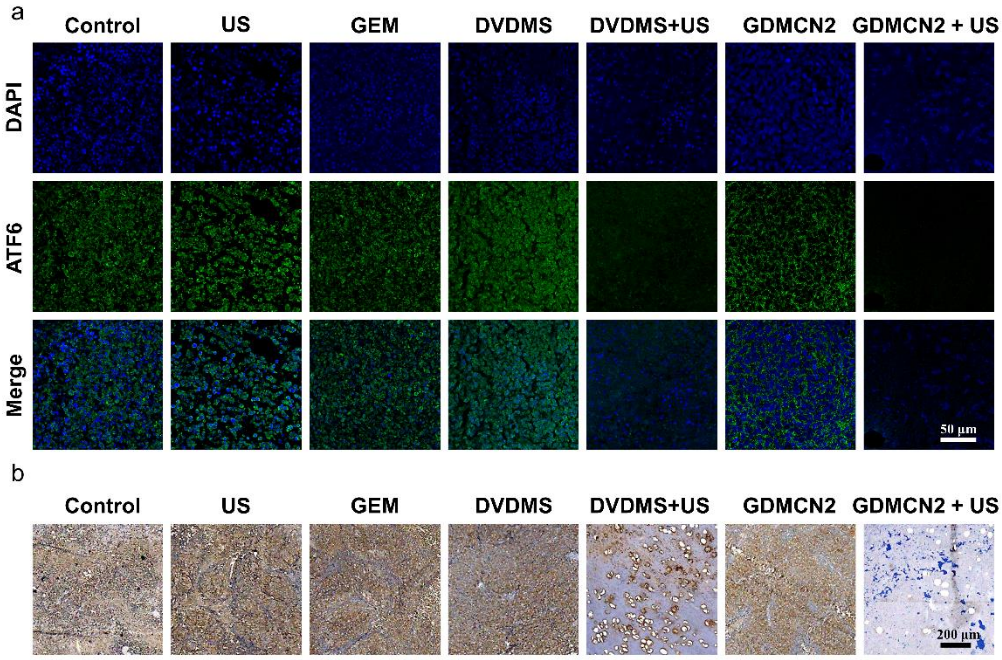

Fig. S25 Images demonstrate TOM20 a) immunofluorescence and b) immunohistochemistry of tumors collected on day 14 of different therapies.

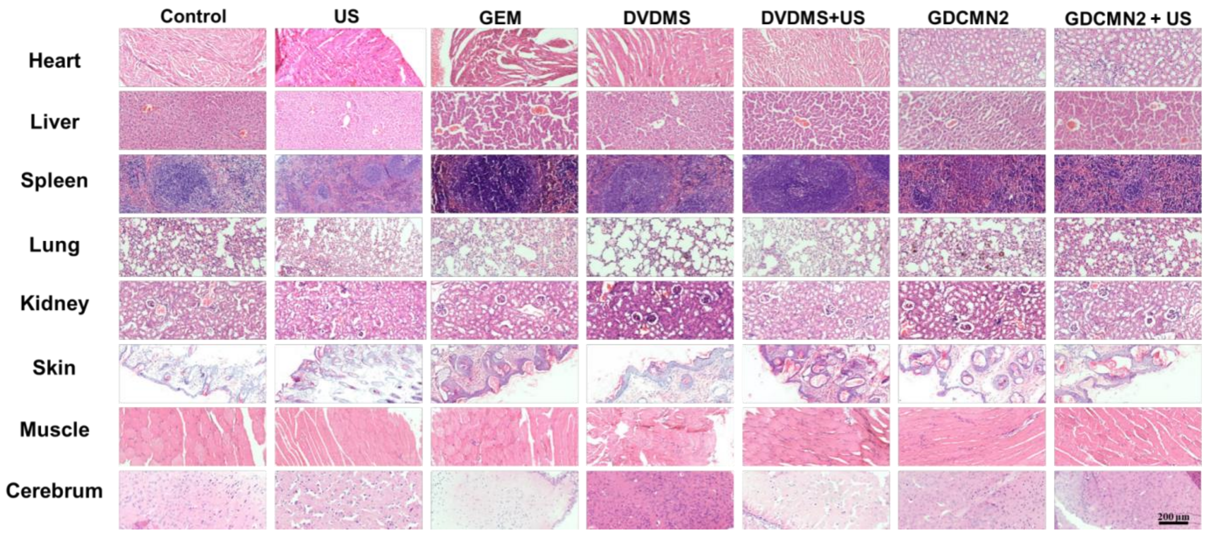

Fig. S26 H&E staining of normal tissues from different treatment group.

Table S1. The EDS element analysis of different samples.

Element	Fe Weight (%)	Fe Atom (%)
NH_2-MIL-101 Fe	31.66	10.11
NH_2-MIL-101(Fe)@TpPa-1-1	9.22	2.37
NH_2-MIL-101(Fe)@TpPa-1-2	5.70	1.41
NH_2-MIL-101(Fe)@TpPa-1-3	2.79	0.67
NH_2-MIL-101(Fe)@TpPa-1-4	2.00	0.48

Table S2. The weight of the nude mice

Markers Weight (g) Time	**DAY 0**	**DAY 2**	**DAY 4**	**DAY 6**	**DAY 8**	**DAY 10**	**DAY 12**	**DAY 14**
(I)-1	17.8	17.9	17.9	16.3	17.8	18.6	18.5	18.6
(I)-2	19.7	19.7	20.1	19.9	19.3	19.7	20.1	20
(I)-3	19.3	19.3	16	17.3	19.2	20	19.9	19.8
(I)-4	21.3	21.2	21.2	19	20.2	20.2	20	19.8
(I)-5	20.9	20.9	21.2	20.9	20.8	21.5	21.3	21.2
(II)-1	20.4	20.7	21.2	22.4	22.1	19.6	19.7	19.5

(II)-2	22.6	22.5	22.5	22.2	21.6	21.7	21.5	21.3
(II)-3	20.8	21.4	21.3	21.1	20.7	20.2	21.1	20
(II)-4	19.5	19.7	21.2	20	19.7	19.2	19.6	19.5
(II)-5	19.9	20.2	20.4	20.6	21.6	21.9	21.6	21.4
(III)-1	20.1	20.2	19.9	19.9	20.5	20.3	20.4	20.3
(III)-2	20.2	20.7	20.9	21.4	21.6	21.2	21.2	21.2
(III)-3	21.3	21.4	20.7	20.9	21.3	20.7	20.8	20.7
(III)-4	19.9	19.6	20.1	20.5	20.5	20.4	20.3	20.4
(III)-5	21.1	21.2	21	21.6	21.2	20.9	20.8	20.7
(IV)-1	20.6	21.1	21	21.1	21.3	21.4	21.3	21.2
(IV)-2	19.9	20.5	19.6	19.9	19.3	18.8	18.6	18.6
(IV)-3	16.1	16.5	17	16.8	16.8	16.8	16.8	17
(IV)-4	21.2	21.2	21.3	21.1	20.8	20.7	20.8	20.7
(IV)-5	19	19.3	19.3	19.2	19	19	19.2	19.5
(V)-1	20.1	19.5	19.3	19.2	19.3	19.4	19.6	19.7
(V)-2	19	19.1	18.7	18.6	19	19	18.6	18.5
(V)-3	19.8	19.6	18.6	18.4	20	19.7	19.6	19.6
(V)-4	20	20.1	19.4	19.4	18.2	16.9	16.5	16.4
(V)-5	17.9	20	19.5	18.9	17.8	17.6	17.5	17.4
(VI)-1	19.1	19.6	19.8	18.4	18.8	18.5	18.4	18.3
(VI)-2	19.2	19.7	20.2	19.2	19.3	19.8	19.7	19.5
(VI)-3	18.5	18.4	19.4	19.2	19.9	20.2	20.1	20.2
(VI)-4	20.2	20.3	20.2	20.7	20.7	20.8	20.7	20.6
(VI)-5	17.5	17.8	18.2	18.5	19.1	19.1	19.2	19.2
(VII)-1	19.4	19.5	18.4	17.6	17.7	17.5	17.4	17.5
(VII)-2	21.6	20.3	19.8	19.2	18.8	18.7	18.5	18.6
(VII)-3	20.4	20.5	19.7	19.4	18.6	18.6	18.6	18.5
(VII)-4	20.3	20.7	20.5	20.2	20.8	20.5	20.3	20.1
(VII)-5	18.9	18.9	18.8	18.2	18.8	18.6	18.7	18.7

PANC-1/GEM tumor-bearing nude mice were randomly divided into seven groups: (i) PBS, (ii) US, (iii) GEM, (iv) DVDMS, (V) DVDMS+US, (VI) GDMCN2, (VII) GDMCN2+US.

Table S3. Biochemical blood analysis of the mice treated after different treatments

Markers	WBC (10^9/L)	RBC (10^12/L)	HGB (g/L)	PLT (10^11/L)	MCV (fL)	MCH (pg)	MCHC (g/L)
(I)-1	6.5	7.5	9.5	17.53	4.84	12.6	26.1
(I)-2	6.5	6.21	7.9	18.01	4.78	12.7	26.6
(I)-3	5	6.73	9	25.01	5.1	13.3	26.2
(I)-4	9.7	7.36	9.7	16.53	4.77	13.1	27.6
(I)-5	6.1	7.66	10.2	12.71	4.86	13.3	27.4
(II)-1	10.9	8.03	10.6	11.22	4.85	13.2	27.2
(II)-2	6.5	6.98	9.7	26.12	5.14	13.8	27
(II)-3	6.4	8.41	12.3	32.41	5.26	14.6	27.8
(II)-4	4.5	6.98	9.5	15.03	5.05	13.6	26.9
(II)-5	4.5	7.95	11.2	16.41	5.2	14	27.1
(III)-1	4.5	7.69	11.2	23.71	5.11	14.5	28.5
(III)-2	10	7.18	10	15.44	4.97	13.9	28
(III)-3	5.4	7.91	10.3	29.21	4.83	13	26.9
(III)-4	36	9.53	12.4	7.24	4.48	13	29.1
(III)-5	2.5	8.8	13.2	12.14	5.11	15	29.3
(IV)-1	3	6.81	9.7	5.78	4.98	14.2	28.6
(IV)-2	7.4	8.58	11.9	8.09	4.87	13.8	28.5
(IV)-3	4.4	8.9	13.5	26.46	5.04	15.1	30.1
(IV)-4	5.5	10.58	15	17.22	4.66	14.1	30.4
(IV)-5	4.1	9.52	14.7	12.13	4.99	15.4	30.9
(V)-1	4	8.35	13.5	12.29	5.23	16.1	30.9
(V)-2	3.3	9	13.7	8.45	4.91	15.2	31
(V)-3	5.3	9.53	14	10.09	4.77	14.6	30.8
(V)-4	2.4	9.27	13.4	9.19	4.69	14.4	30.8
(V)-5	4.2	9.1	14.4	8.58	5.27	15.8	30
(VI)-1	3.5	9.33	14.3	18.06	5.06	15.3	30.2
(VI)-2	6	8.43	12.8	16.25	4.93	15.1	30.8
(VI)-3	3.2	8.94	13.2	10.78	4.86	14.7	30.4
(VI)-4	5.4	9.31	13.7	13.01	4.88	14.7	30.1
(VI)-5	3.8	9.12	13.8	10.89	4.97	15.1	30.4
(VII)-1	3.8	9.12	13.8	10.89	4.97	15.1	30.4

(VII)-2	7.2	9.16	13.5	28.03	4.92	14.7	30
(VII)-3	3.4	8.7	13.3	15.45	5.05	15.2	30.2
(VII)-4	2.5	9.08	13.7	21.12	4.93	15	30.6
(VII)-5	7.1	7.28	13.5	12.89	5.84	18.5	31.7

PANC-1/GEM tumor-bearing nude mice were randomly divided into seven groups: (i) PBS, (ii) US, (iii) GEM, (iv) DVDMS, (V) DVDMS+US, (VI) GDMCN2, (VII) GDMCN2+US.
